# A High-Level Language for Rule-Based Modelling

**DOI:** 10.1371/journal.pone.0114296

**Published:** 2015-06-04

**Authors:** Michael Pedersen, Andrew Phillips, Gordon D. Plotkin

**Affiliations:** 1 Department of Plant Sciences, Cambridge University, Cambridge, England; 2 Biological Computation Group, Microsoft Research, Cambridge, England; 3 Laboratory for Foundations of Computer Science, School of Informatics, Edinburgh University, Edinburgh, Scotland; Leibniz Institute for Age Research, GERMANY

## Abstract

Rule-based languages such as Kappa excel in their support for handling the combinatorial complexities prevalent in many biological systems, including signalling pathways. But Kappa provides little structure for organising rules, and large models can therefore be hard to read and maintain. This paper introduces a high-level, modular extension of Kappa called LBS-*κ*. We demonstrate the constructs of the language through examples and three case studies: a chemotaxis switch ring, a MAPK cascade, and an insulin signalling pathway. We then provide a formal definition of LBS-*κ* through an abstract syntax and a translation to plain Kappa. The translation is implemented in a compiler tool which is available as a web application. We finally demonstrate how to increase the expressivity of LBS-*κ* through embedded scripts in a general-purpose programming language, a technique which we view as generally applicable to other domain specific languages.

## Introduction

Mathematical modelling plays a key role in systems biology, facilitating the generation of knowledge through the cycle of model analysis, experimental testing of hypotheses, and model refinement. As our biological knowledge base increases through improvements in experimental techniques, the models under study also increase in size and complexity. Large models based on traditional mathematical formalisms such as ODEs are hard to develop and maintain since they are “flat” with no structure or modularity and since they bear a somewhat indirect relation to the corresponding biological phenomena. This has prompted the development of a range of new modelling formalisms inspired by computer science, allowing models to be written in a structured fashion and generally supporting multiple types of simulation and analysis based on a single model. These formalisms include process calculi such as the stochastic *π*-calculus [1, 2], the continuous *π*-calculus [3], Beta binders [4, 5], BlenX [6], PEPA [7] and BioPEPA [8]; and rule-based languages such as BIOCHAM [9], Kappa [10, 11], BioNetGen [12] and Stochastic Multilevel Multiset Rewriting [13].

Some of the above formalisms, in particular those based on process calculi, have built-in support for modularity, allowing large systems to be described in terms of their components. Other such formalisms, however, lack modular features, complicating the development and maintenance of large models. Higher-level modular extensions have thus been developed on top of these formalisms. The Language for Biochemical Systems (LBS) [14, 15] is one such extension, defined on top of standard biochemical reactions. But LBS is more than an extension: it is a general framework which is parameterised on key structures, allowing for instantiation to other languages.

In this paper we show how LBS can be instantiated to yield a modular extension of the otherwise flat rule-based language Kappa. Rules in Kappa describe the transformation of complexes at the level of *agent* (protein) binding sites, effectively handling the combinatorial explosion in the number of species, which is a key problem in the modelling of signalling pathways in particular. The instantiation of LBS to Kappa, called LBS-*κ*, can be adapted with minor modifications to the closely related BioNetGen rule-based language. The contribution of the instantiation is two-fold: providing a language for writing modular rule-based models and also demonstrating the generality of LBS as a framework. As an additional contribution, a tool for LBS-*κ* has been implemented and is available as a web application directly through a browser. The tool provides a compiler from LBS-*κ* to flat Kappa, and it provides a simulator for the resulting flat models.

As a brief illustration, the following LBS-*κ* model shows how a generic phosphorylation module can be defined and instantiated. The module takes two parameters, namely an enzyme a with binding site m, and a substrate b with binding site n. The module consists of three rules: the first for binding the agents on their given sites as indicated by the binding label 1, the second for phosphorylating the substrate, and the third for unbinding. Phosphorylation states are indicated by u and p, meaning respectively unphosphorylated and phosphorylated. The first two rules apply only when the substrate is unphosphorylated, whereas the third applies in either case. All three rules apply regardless of any enzyme modification state, and regardless of any enzyme binding on other sites than the one given. The instantiation provides the parameter Raf as the enzyme and MEK as the substrate.

1 **module** phosphorylate(**agent** a{m}, b{n}){

2  a{m} + b{n˜u} → a{m! 1}-b{n˜u! 1}|

3  a{m! 1}-b{n˜u! 1} → a{m! 1}-b{n˜p! 1}|

4  a{m! 1}-b{n! 1} → a{m} + b{n}

5 };

6

7 phosphorylate(Raf{x}, MEK{S222})

The LBS framework is designed with formal foundations in mind, based on the modular Calculus of Chemical Systems [16]. While formal foundations may be of little interest to the general modelling practitioner, there are some key advantages. One is that the language itself is precisely defined, so there is no ambiguity in the meaning of any of the language constructs; the intended meaning can be obtained by reference to the formal semantics. A second is that new instantiations of the framework, such as LBS-*κ*, can be defined with relative ease, without having to redefine the full language. And a third advantage is that key properties of an LBS instantiation can be studied independently of the full language, as was demonstrated in [17] for Petri net invariants. In the Kappa context, this may for example prove useful in studies of modular analysis techniques.

In addition to the modular features inherited from the Calculus of Chemical Systems, LBS provides features such as compartments, complex agent expressions, combinatorial combinators, and parameterised modules with a notion of agent subtyping. In the Results section we first introduce these features informally through examples. We then demonstrate through case studies of a chemotactic switch ring, a MAPK cascade, and an insulin signalling pathway, how the features of LBS-*κ* give rise to improved readability, maintainability and reusability. We finally introduce the LBS-*κ* web application, and we demonstrate how the expressive power of LBS-*κ* can be increased by introducing embedded scripts written in the general purpose programming language F#. In the Discussions section we consider related work with a particular focus on two different approaches to high-level language development: one via domain-specific languages such as LBS-*κ*, and the other via embedded languages such as the recent PySB [18] which is based on the general-purpose language Python. In the Methods section we formally define the LBS-*κ* language. Since LBS is a general framework, we need only define the necessary instantiation to Kappa, and refer to the previously published general semantics of LBS [15]. The Methods section assumes an understanding of basic computer science, but the remainder of the paper can be read independently of this section.

## Results

### 2.1 Language Overview

#### 2.1.1 Agents, Rules, Composition and Compartments

An *agent expression* describes one or more species. For example, Raf {x˜p} is an atomic agent with name Raf that is phosphorylated on site x. More generally, *post-translational modification* is represented by an internal state name following a tilde symbol; we write p for phosphorylated and u for unphosphorylated. Agents can be composed into complexes using the *agent composition* operator, −; for example Raf {x˜p! 1}−MEK {S222˜u! 1} describes a complex of two agents, Raf and MEK. The *binding* is represented by the label 1 following an exclamation mark on the respective sites. The label can be any integer, but must distinguish a particular binding from any other bindings that may be present in a rule.

A *rule* describes the transformation of agents at the level of binding sites. Take for example the following rule which expresses the binding of two proteins, Raf and MEK, on their respective sites x and S222.

1 Raf{x˜p} + MEK{S222˜u} → Raf{x˜p! 1}-MEK{S222˜u! 1}

The left hand side of the rule thus specifies that for the rule to apply, Raf must be phosphorylated on x and MEK must be unphosphorylated on S222. The respective internal states are the same on the left hand side (LHS) and the ride hand side (RHS) and are hence preserved by the rule. The absence of any binding labels on the LHS states that the agents cannot be bound on their respective sites before applying the rule. But the rule imposes no further conditions; in particular, the rule can be applied in situations where RAF or MEK are bound to other proteins on other sites, regardless of the internal state of any other sites. In this way a rule generally represents many concrete reactions, thus effectively helping alleviate the problem of combinatorial explosion of the size of models.

We have adopted a syntax for rules which differs slightly from both Kappa and BioNetGen in order to accommodate the higher-level language constructs of LBS-*κ*. In particular, sites are specified within curly brackets, {}, rather than within parentheses. We explicitly represent complexes using the dash symbol, −, and we represent distinct agents within a rule by separation with the + symbol. This syntax follows the BioNetGen convention, whereas Kappa syntax instead has a flat structure of comma-separated agents. Note that rules can generally be equipped with rates, which are written in LBS-*κ* in brackets following arrows as in e.g. A → {0.1} B. We omit rates from the examples for the sake of clarity.

While agents often model proteins they can also represent other objects, for example chemical compounds such as nucleic acids. The following is a high-level example of MEK gene expression in which mRNA is transcribed from a gene and an RNA polymerase rnap in the first rule, and the mRNA is translated into MEK in the second rule.

1 gene + rnap → gene + rnap + mRNA|

2 mRNA → MEK

Observe how the two rules are composed using the *parallel composition* operator, ∣, which is the glue used more generally for joining two models together. In this example the rules correspond to standard, concrete reactions, given that none of the participating reactant agents have any sites.

The *location operator*, [], allows models to be composed within a tree-structured hierarchy of static compartments, and furthermore allows for cross-compartment transport rules. The following example shows how the above binding example and gene expression example can be composed within a compartment structure. Gene expression now takes place within the nucleus compartment, and we have added an additional rule for transporting mRNA out of the nucleus into the top level cell compartment before translation. Note that multiple occurrences of the same compartment name refer to the same compartment.

1 cell[

2  Raf{x˜p} + MEK{S222˜u} → Raf{x˜p! 1}-MEK{S222˜u! 1}|

3  nucleus[

4   gene + rnap → gene + rnap + mRNA

5  ]|

6  nucleus [mRNA] → mRNA|

7  mRNA → MEK{S222˜u}

8 ]

#### 2.1.2 Agent Expressions for Complexes

The rules in the previous section are relatively short and easy to read. However, larger rules containing many agents can be hard to read, as demonstrated by the following two rules adapted from a model in [10]. The first rule uses the *wild card* binding symbol _, meaning that the site must be bound to some unspecified agent. It expresses phosphorylation of Shc. The second rule expresses binding of Ras to a large complex.

1 EGFR{CR!_, Y1148˜p! 1}-Shc{PTB! 1, Y318˜u} → EGFR{CR!_, Y1148˜p! 1}-Shc{PTB! 1, Y318˜p}|

2

3 EGFR{Y1148˜p! 1}-Shc{PTB! 1, Y318˜p! 2}-Grb2{SH2! 2, SH3! 3}-SoS{a! 3, b} + Ras{S1S2˜gdp} →

4  EGFR{Y1148˜p! 1}-Shc{PTB! 1, Y318˜p! 2}-Grb2{SH2! 2, SH3! 3}-SoS{a! 3, b! 4}-Ras{S1S2˜gdp! 4}

We use an explicit notation for complexes to simplify reading as compared to the standard Kappa rules. Despite this simplification, it still remains difficult at first glance to determine what *actions* the second rule expresses. Doing so requires a detailed comparison of the LHS to the RHS. LBS-*κ* addresses this through a richer notion of agent expressions by allowing complexes to be abbreviated and subsequently updated. The above rules can then be re-written as follows:

1  **agent** c1 = EGFR{CR!_, Y1148˜p! 1}-Shc{PTB! 1, Y318˜u};

2 c1 → c1〈Shc{Y318˜p}〉|

3

4 **agent** c2 = EGFR{Y1148˜p! 1}-Shc{PTB! 1, Y318˜p! 2}-Grb2{SH2! 2, SH3! 3}-SoS{a! 3, b};

5 c2 + Ras{S1S2˜gdp} → c2〈SoS{b! 4}〉-Ras{S1S2˜gdp! 4}

In both cases the LHS components are assigned to an identifier, c1 and c2, respectively. The identified complex is then referred to and updated on the RHS of the rules using the *update operator*, 〈〉. The update for the first rule states that the RHS is exactly the same as c1, except that species Shc is phosphorylated on site Y318. The update for the second rule states that the RHS is a complex obtained from c2 by adding a link from Sos to Ras.

As a further abbreviation, agent expressions can be defined *in-line* using the **as** keywords, as follows:

1 EGFR{CR!_, Y1148˜p! 1}-Shc{PTB! 1, Y318˜u} **as** c1 → c1〈Shc{Y318˜p}〉|

2

3 EGFR{Y1148˜p! 1}-Shc{PTB! 1, Y318˜p! 2}-Grb2{SH2! 2, SH3! 3}-SoS{a! 3, b} **as** c2 + Ras{S1S2˜gdp} →

4  c2〈SoS{b! 4}〉-Ras{S1S2˜gdp! 4}

#### 2.1.3 New Agents

All agents must be defined using the *new operator* before first use, although we have omitted doing so in the preceding examples. The Shc agent can for example be defined as follows:

1 **agent** c = **new** Shc{PTB:(u p), Y318:(u p)};

The **new** operator specifies the name of the new agent together with the internal state values each site can take. Internal state values default to u and p, so could have been omitted in the above example. As the name suggests, the **new** operator does indeed generate a new agent, with a name Shc, that is universally unique within the model. If the above definition were repeated, the two occurrences of the c identifier would bind to semantically distinct agents. However, the identifier is significant because it is needed for the update expression shown in the previous example, i.e. c 〈Shc {Y318˜p}〉. Often it is convenient for the agent identifier to which a new agent is assigned to be the same as the agent name, as follows:

1 **agent** Shc = **new** Shc{PTB:(u p), Y318:(u p)};

In these cases we can omit the agent name on the right hand side as an abbreviation, as follows:

1 **agent** Shc = **new** {PTB:(u p), Y318:(u p)};

We use a similar abbreviation for updates on atomic agents, so that e.g. Shc {Y318˜p} in fact is an abbreviation for the expression Shc 〈Shc{Y318˜p}〉.

#### 2.1.4 Parameterised Modules

A key aspect of high-level languages is their support for modularity. Modules in LBS-*κ* can be parameterised on agents and their sites; on compartments; and on rates. The following is a verbose example of a module encapsulating phosphorylation through three rules, namely one for binding, a second for phosphorylation, and a third for unbinding:.

1 **module** phosphorylate(**agent** c1:k{m}, c2:s{n}){

2  c1〈k{m}〉 + c2〈s{n˜u}〉 → c1〈k{m! 1}〉-c2〈s{n˜u! 1}〉|

3  c1〈k{m! 1}〉-c2〈s{n˜u! 1}〉 → c1〈k{m! 1}〉-c2〈s{n˜p! 1}〉|

4  c1〈k{m! 1}〉-c2〈s{n! 1}〉 → c1〈k{m}〉 + c2〈s{n}〉

5 };

6

7 **agent** r = **new** Raf{x, y};

8 **agent** m = **new** MEK{t, S218, S222};

9 phosphorylate(r:Raf{x}, m:MEK{S222})

The first formal parameter in line 1, **agent** c1: k {m}, is for the kinase. It states that an agent expression is expected with at least one atomic kinase agent containing at least one site. Given the corresponding actual parameter r: Raf {x} in the module invocation in line 9, the identifier c1 is bound to the same expression as r is bound to at module invocation time, which happens to be an atomic agent expression; the agent name k is bound to the agent name Raf; and the site name m is bound to the site name x. Observe how agent Raf has more sites than are required by the module, which is fine as long as it has *at least* the number of required sites, and with matching types (in the above, the default site types are assumed in both formal and actual parameters). This hence corresponds to a notion of subtyping. A similar notion applies at the level of agent expressions: the identifier r could have been bound to a complex species in the module invocation. For the sake of illustration, the modular phosphorylation model “expands” to the following equivalent flat model:

1 **agent** Raf = **new** {x, y};

2 **agent** MEK = **new** {t, S218, S222};

3

4 Raf{x} + MEK{S222˜u} → Raf{x! 1}-MEK{S222˜u! 1}|

5 Raf{x! 1}-MEK{S222˜u! 1} → Raf{x! 1}-MEK{S222˜p! 1}|

6 Raf{x! 1}-MEK{S222! 1} → Raf{x} + MEK{S222}

Following our abbreviations for atomic agent expressions outlined in Section 2.1.3, the phosphorylation module can be simplified because it only deals with atomic agents. Hence there is no need to distinguish the formal agent name and the corresponding identifier, so the formal parameter **agent** c1: k{m} could just as well be written as **agent** k: k {m} which is abbreviated simply as **agent** k: {m}. With the corresponding abbreviations in the body of the module, the phosphorylation example can be written more concisely as follows:

1 **module** phosphorylate(**agent** k:{m}, s:{n}){

2  k{m} + s{n˜u} → k{m! 1}-s{n˜u! 1}|

3  k{m! 1}-s{n˜u! 1} → k{m! 1}-s{n˜p! 1}|

4  k{m! 1}-s{n! 1} → k{m} + s{n}

5 };

6

7 **agent** Raf = **new** {x, y};

8 **agent** MEK = **new** {t, S218, S222};

9 phosphorylate(Raf:{x}, MEK:{S222})

#### 2.1.5 Agent Aliases

Sometimes we need to be able to identify and update one of several agents with the same name within a complex. Agent *aliases* can be created in conjunction with the **new** operator for this purpose. Consider the following example of a rule from the chemotactic switch ring model case study involving a homo-multimer containing three agents with the same name P; internal states here take values 0 and 1.

1 **agent** P1, P2, P3 = **new** P{f:(0 1), x:(0 1), y:(0 1), s};

2

3 **agent** c000 = P1{f˜0, y! 1}-P2{x! 1, f˜0, y! 2, s}-P3{x! 2, f˜0};

4 c000 ↔ c000〈P2{f˜1}〉

Aliases P1, P2 and P3 are created to allow the rule to update only a selected agent, namely the second one, within the complex. This example also shows the use of reversible rules using the bidirectional arrow, ↔.

#### 2.1.6 Non-determinism

Another feature of agent expressions is *non-determinism*, which provides a means of grouping agents which are functionally similar through the **or** operator. The following example, adapted from [11], shows how the binding between a family of ERK and MEK proteins can be expressed using this mechanism:

1  **agent** M = MEK1 **or** MEK2;

2  **agent** E = ERK1 **or** ERK2;

3 M〈MEK{D}〉 + E〈ERK{CD}〉 → M〈MEK{D! 1}〉-E〈ERK{CD! 1}〉

The first line states that MEK can either be MEK1 or MEK2, and the second line is analogous for ERK. The third line expresses binding between these families of proteins variants; it expands to the following four concrete rules, one for each combination of proteins within the two families:

1 MEK1{D} + ERK1{CD} → MEK1{D! 1}-ERK1{CD! 1}|

2 MEK1{D} + ERK2{CD} → MEK1{D! 1}-ERK2{CD! 1}|

3 MEK2{D} + ERK1{CD} → MEK2{D! 1}-ERK1{CD! 1}|

4 MEK2{D} + ERK2{CD} → MEK2{D! 1}-ERK2{CD! 1}

For this example to work, the agents MEK1 and MEK2 must initially be defined to allow updates on the same site D on a common agent name MEK, and similarly for ERK1 and ERK2. This is achieved by passing the common name to the **new** operator as follows:

1 **agent** MEK1 = **new** MEK{D, S218, S222};

2 **agent** MEK2 = **new** MEK{D, S222, S226};

3 **agent** ERK1 = **new** ERK{CD, T202, Y204};

4 **agent** ERK2 = **new** ERK{CD, T185, Y187};

### 2.2 Case Studies

#### 2.2.1 The Chemotactic Switch Ring

**Fig 1:**
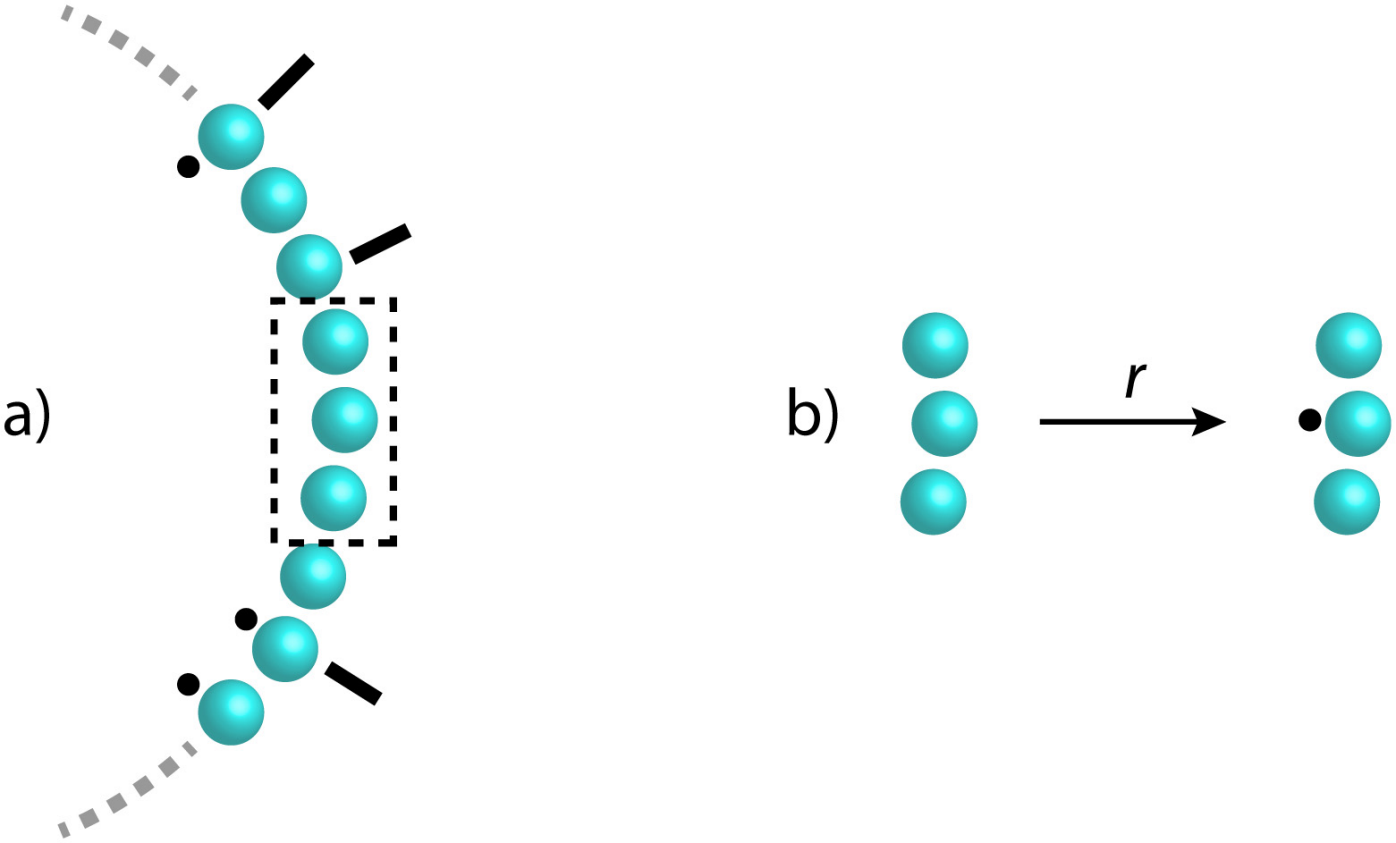
The chemotactic switch ring. An illustration of the chemotactic switch ring (a) and the activation reactions taking place within the ring (b). The black dots indicate the active state, and the black lines indicate the bound state. The reaction rate depends on both the active state and the binding state of the neighbouring protomers.

Chemotaxis is the process whereby bacteria move in their environment, generally towards nutrients or away from toxins. Some bacteria, including *Escherichia coli*, achieve such movement through the mechanical rotation of *flagella* attached to the cell surface. The direction of flagella rotation is determined by a switch consisting of a ring of 34 protomers each with two conformations, active and inactive, which collectively determine the overall switch state of the ring [19, 20]. Each protomer can switch from inactive to active conformations, and the switch is catalysed by either a binding to a phosphorylated CheY protein or by neighbouring protomers being active. Fig 1 illustrates the ring conformation and the activation reactions.

We here consider a flat Kappa model by Vincent Danos of the chemotactic switch ring, specifically a subset which captures the modular structure of interest. The subset is reproduced in S1 Listing with permission from the author. It consists of 8 reversible rules, each expressing conformational change of a protomer within a context of two neighbour protomers. A protomer P has four sites: two for binding in the ring (x and y), one for binding CheY (s), and one for representing conformational state (f) where the internal state 0 represents inactive and 1 represents active. The full model additionally includes rules for CheY binding and initial conditions which are not relevant for our case study. The initial conditions, which define a ring with 34 protomer agents, are revisited in Section 2.3.2 in the context of embedded scripts.

An LBS-*κ* version of the flat model in S1 Listing is shown in (Table 1). We use identifiers of the form cXXX to represent agent expressions with three protomers, with the values of the Xs indicating the conformational state of each. The bulk of the model lies in the definition of a module called flips, which contains four rules for conformational changes in each of the possible states of neighbour conformation. The module is parameterised on a complex with three protomers, with the middle one being subject to conformational change. Note that the parameters include site types in addition to site names, so e.g. P2{f:(01)} refers to an agent P2 with a site f that can take values 0 or 1. The module is also parameterised on a forward and reverse rate multiplier, *k*. Within the flips module an additional, nested module called flip is defined, which embodies a single-rule flip. Defining a single-rule module may not reduce the size of the model, but it does add clarity by naming a repeated pattern.

**Table 1:** Some of the symbols used in LBS-*κ* syntax and semantics.

**Symbol**	**Description**
*id*_c_	Compartment identifier
*e*_a_	Agent expression
*v*_s_	Agent value
*id*_a_	Agent identifier
*n*_a_	Agent name
*v*_s_	Site name
*ξ*	Agent annotation
*R*	Normal form rule
*v*_na_	Normal form agent value

**Listing 1:** An LBS-*κ* version of the chemotactic switch ring.

1 **module** flips(**agent** c000: P1{f: (0 1)}-P2{f: (0 1)}-P3{f: (0 1)}; rate k) 2 { 3 **module** flip(**agent** cxxx:P{f:(0 1)}; **rate** r1; **rate** r2) { 4 cxxx〈P{f˜0}〈 ↔{r1*k}{r2} cxxx〈P{f˜1}〉 5 }; 6 7 **agent** c100 = c000〈P1{f˜1}〉; 8 **agent** c001 = c000〈P3{f˜1〉; 9 **agent** c101 = c000〈P1{f˜1}〉〈P3{f˜1}〉; 10 11 flip(c000:P2{f}, 1, 200)| 12 flip(c100:P2{f}, 1, 2)| 13 flip(c001:P2{f}, 1, 2)| 14 flip(c101:P2{f}, 100, 2) 15 }; 16 17 **agent** P1, P2, P3 = **new** P{f:(0 1), x:(0 1), y:(0 1), s}; 18 **agent** c000 = P1{f˜0, y! 1}-P2{x! 1, f˜0, y! 2, s}-P3{x! 2, f˜0}; 19 **agent** c000b = c000〈P2{s!_}〉; 20 21 flips (c000:P1{f}-P2{f}-P3{f}, 1)| 22 flips (c000b:P1{f}-P2{f}-P3{f}, 10)

Two instantiations of the module are composed in the last two lines: in the second instantiation the middle protomer is bound to CheY, and in the first it is unbound. The binding to CheY is expressed implicitly through a wild card, which is adequate here since protomers never bind other agents on this site. Observe how the rate multipliers differ by an order of magnitude in the two cases. Observe also how the module instantiations make explicit the subtle difference between the two blocks of flip rules, which would not otherwise be immediately apparent from a flat model.

The model demonstrates the use of agent updates and the use of modules parameterised on agents and rates. Observe that the flips module only specifies a single site for each protomer, while the agents in instantiations have multiple sites, demonstrating site subtyping. The nested flip module only specifies a single agent in its parameter, while the instantiations provide complexes but with the relevant agent of each complex specified; this demonstrates subtyping at the higher level of complexes.

#### 2.2.2 A MAPK Cascade

**Fig 2:**
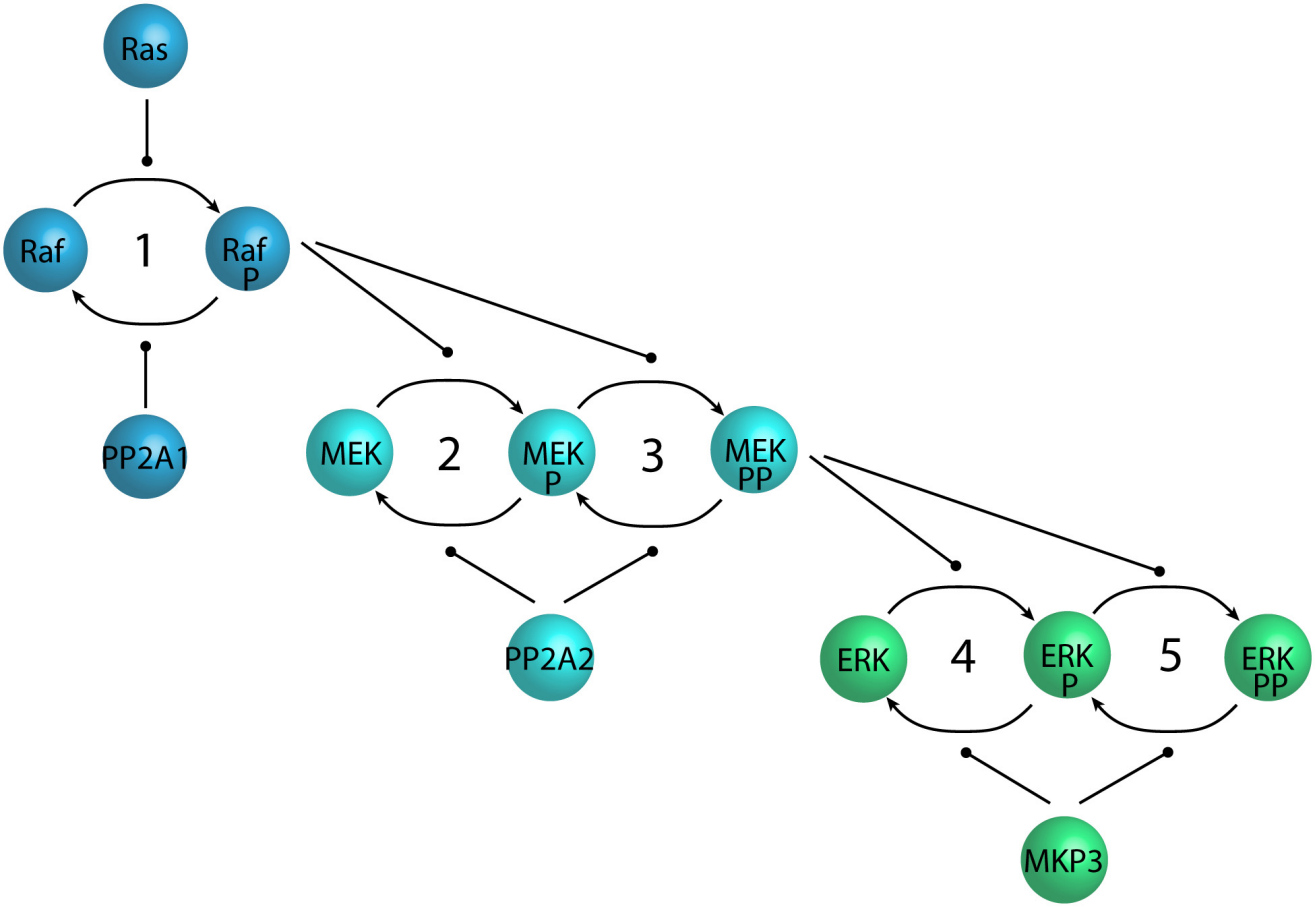
The MAPK cascade. An illustration of the MAPK cascade, amplifying a signal through three layers of protein modification. The lines with circular heads indicate catalysis, and the Ps indicate phosphorylation.

The Mitogen-Activated Protein Kinase (MAPK) cascade is a common motif in signalling pathways, and has the functional property of amplifying an upstream signal through three layers of protein modification as illustrated in Fig 2. The first layer has a single phosphorylation/dephosphorylation cycle, and the two subsequent layers each have two cycles involving two different sites of the same substrate protein. A simple Kappa model of a MAPK cascade is given in [21]. It is reproduced with permission from the author in S2 Listing with some restructuring to highlight the underlying modularity. The model represents each cycle by six rules: three for phosphorylation, and three for dephosphorylation.

An LBS-*κ* version of the model is shown in (Table 2). The model defines a cycle module in lines 1-16 which is parameterised on a kinase (k), a phosphatase (p) and a substrate (s); all three species parameters specify just one site, all called m. The module defines two sub-modules, one for binding, phosphorylation and unbinding (phos) which is parameterised on the kinase and the substrate, and one for binding, dephosphorylation and unbinding (dephos) which is parameterised on the phosphatase and substrate. Note how in-line agent definitions are used to abbreviate the substrate complexes: the *identity updates* e on the RHS of lines 5 and 11 result in the binding state remaining unaffected, i.e. bound in these cases. The body of the cycle module simply consists of instantiations of the two sub-modules in parallel.

**Table 2:** The abstract syntax for general LBS agent expressions, parameterised on site types *ρ* and site expressions *e*_s_.

*e*_a_	∷=		Agent expression
	∣	**new** *n*_a_{*σ*}	New agent
	∣	*id*_c_[*e*_a_]	Location
	∣	$e_{\text{a}}-e_{\text{a}}^{\prime }$	Composition
	∣	$e_{\text{a}}\langle \underline{{\text{id}}_{\text{c}}}[n_{\text{a}},\alpha ]\rangle$	Update
	∣	$e_{\text{a}}\;\text{or}\;e_{\text{a}}^{\prime }$	Choice
	∣	*e*_a_ : *ξ*	Annotation
	∣	*id*_a_	Identifier
*ξ*	∷=	$\;\;\underline{\underline{{\text{id}}_{\text{c}}}[n_{\text{a}},\;\underline{n_{\text{s}}}]}$	Annotation
*σ*	∷=	{*n*_s_ ↦ *ρ*}	Agent type
*α*	∷=	{*n*_s_ ↦ *e*_s_}	Site assignments

**Listing 2:** An LBS-*κ* version of the MAPK cascade from [21].

1 **module** cycle(**agent** k:{m}, p:{m}, s:{m}){ 2 3 **module** phosphorylate(**agent** k:{m}, s:{m}){ 4 k{m} + s{m˜u} → k{m! 1}-s{m˜u! 1} **as** c; 5 c ˜ c〈s{m˜p! e}〉| 6 k{m! 1}-s{m˜p! 1} → k{m} + s{m˜p} 7 }; 8 9 **module** dephosphorylate (**agent** p: {m}, s: {m}){ 10 p{m} + s{m˜p} → p{m! 1}-s{m˜p! 1} **as** c; 11 c → c〈s{m˜u! e}〉| 12 p{m! 1}-s{m˜u! 1} → p{m} + s{m˜u} 13 }; 14 15 phosphorylate (k: {m}, s: {m})|dephosphorylate(p: {m}, s: {m}) 16 }; 17 18 **agent** Ras = **new** {n: (gtp gdp)}; 19 **agent** Raf = **new** {n}; 20 **agent** MEK = **new** {S218, S222, n}; 21 **agent** ERK = **new** {T185, Y187}; 22 **agent** PP2A1 = **new** {n}; 23 **agent** PP2A2 = **new** {n}; 24 **agent** MKP3 = **new** {n}; 25 26 cycle (Ras: {n}, PP2A1: {n}, Raf: {n})| 27 cycle (Raf{n˜p}: {n}, PP2A2: {n}, MEK: {S218})| 28 cycle (Raf{n˜p}: {n}, PP2A2: {n}, MEK: {S222})| 29 cycle (MEK{S218˜p, S222˜p, n}: {n}, MKP3: {n}, ERK: {T185})| 30 cycle (MEK{S218˜p, S222˜p?, n}: {n}, MKP3: {n}, ERK: {Y187})

Lines 18-24 define the agents used in the model. All agents have the default internal state types (i.e. u and p) except for Ras which therefore explicitly declares the type for its site (gdp and gtp). Lines 26-30 consist of a parallel composition of five module invocations, one for each cycle. Note the subtle distinction between the state of parameter agents and the site parameter: in e.g. line 29, the MEK parameter is phosphorylated on sites S218 and S222 and can have any internal state on site n, but it is the latter site n which is passed to the module for binding.

**Fig 3:**
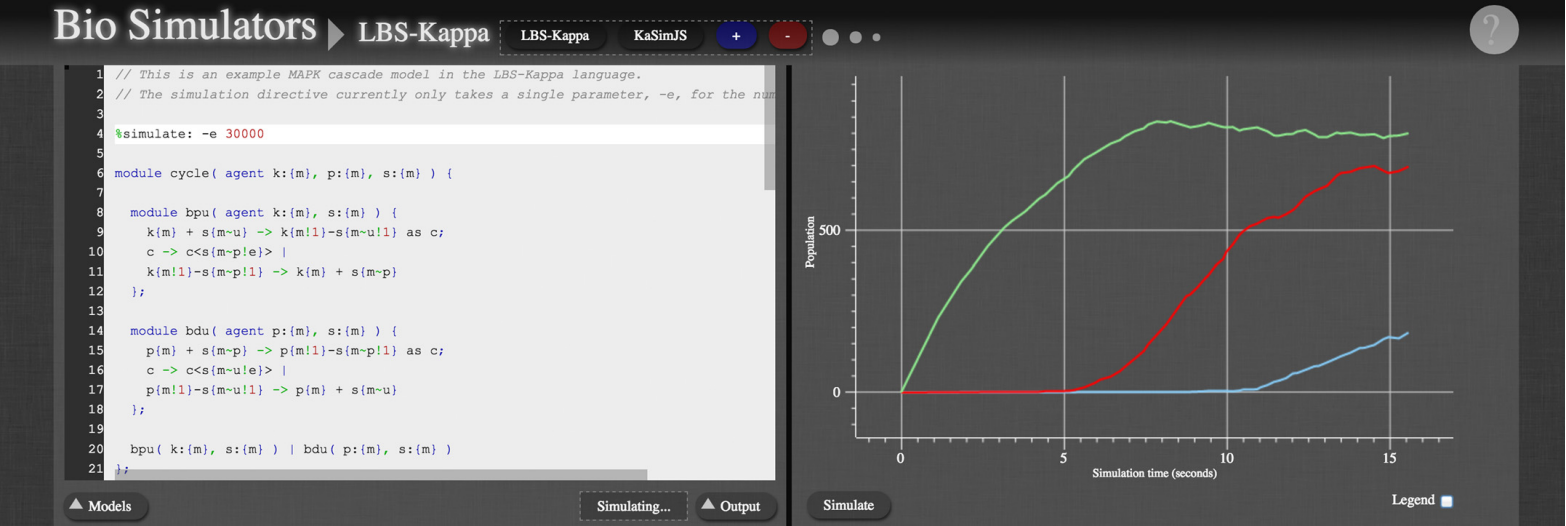
A screenshot of the web application for LBS-*κ*. The left hand side provides a syntax-highlighting editor, and the right hand side shows time course simulation plots.

This example demonstrates the use of parameterised modules with multiple instantiations and with site subtyping. The outer module effectively has six rules and five instantiations, and the inner modules each have three rules and a single instantiation. In addition to clarifying the model through structure, the model is also significantly smaller than the corresponding flat model with 30 rules. Note finally that the inner modules are very general, and could hence be defined in a standard library for wider use as in e.g. [18]. A simulation plot for the MAPK cascade model is shown in Fig 3 in the context of the web tool discussed in Section 2.3.

#### 2.2.3 Insulin Signalling

The insulin signalling pathway responds to rises in blood glucose levels and accompanying rises in insulin levels with the net effect of inducing glucose uptake by cells. Numerous models have been developed using traditional mathematical formalisms which are challenged by the combinatorial complexity arising from e.g. receptor-ligand binding [22, 23]. We here consider a Kappa model of insulin signalling by Isha Antani and Gordon Webster. The model is reproduced in S3 Listing with permission from the authors and contains approximately 120 rules, listed with no clear a priori modular structure; it hence constitutes a challenging test case for LBS-*κ*. The full LBS-*κ* version of the model is given in S4 Listing.

The LBS-*κ* model exploits modularity at two levels. At the top level are modules with no parameters and only one instance each. These are used purely for structure, allowing the main body of the model to be specified as a parallel composition of module instantiations, each representing a functionally self-contained component:

1 receptorActivation() | pipAktSignalling() | gluconeogenesis() |

2 glycogenSynthesis() | cellGrowth()     | glucoseUptake()

At the second level are modules with multiple instances. They are all small, containing up to three rules, many of which represent variations of the phosphorylation (bpu) and dephosphorylation (bdu) modules used in the MAPK cascade; these variations are in turn defined as instances of more general phosphorylation and dephosphorylation modules which are parameterised separately on agents for each of the three rules, allowing agent states to differ both between rules and between instances. Some modules contain just a single rule, e.g. for binding, unbinding and modification. Even though the resulting decrease in model size is inconsequential, these small modules do result in increased clarity by directly exposing the *actions* associated with one or more rules; they also implicitly expose rules which confer more complicated actions not embodied in standard modules. Consider the following excerpt:

1 bpu3(PDK1{PH}:{PH, pkc}, PKCz:{T410, pdk1})|

2

3 Akt{as160, S473˜p, T308˜p} + AS160{gap˜u! 1}-Rab10{g˜u! 1} →

4  Akt{as160! 1, S473˜p, T308˜u}-AS160{gap˜u! 1} + Rab10{g˜u}|

5 pho(Akt{as160! 1}-AS160{gap˜u! 1}: AS160{gap})|

6 unbind(Akt:{as160}, AS160:{gap})

The first line immediately conveys that PDK1 phosphorylates PKCz through some standard mechanism embodied by the bpu3 module. The following three lines immediately convey that Akt phosphorylates and unbinds AS160 through the pho and unbind modules (lines 5 and 6), but also that the binding rule (lines 3 and 4) is more complicated and may require further examination. Of course this approach relies on meaningful naming conventions for modules; further tool support could be helpful here, e.g. by providing a shortcut for going to the definition of a module from a module instantiation point.

The LBS-*κ* model uses non-determinism in two cases, namely for the related GSK3a and GSK3b agents, and for the related IRS1 and IRS2 agents. It also uses agent expressions in several cases, e.g. for reducing the complexity of long rules as follows:

1 Akt{PH! 1, T308˜u}-PIP{three˜p! 1} **as** a +

2  PDK1{PH! 2, akt}-PIP{three˜p! 2} **as** b →

3  a〈Akt{T308! 4}〉-b〈PDK1{akt! 4}〉 **as** c;

4 c → c〈Akt{T308p˜! e}〉

This last rule exploits the identity update feature also used by the MAPK cascade: Akt {T308˜p! e} expresses that the site T308 should be phosphorylated, but that the binding state should remain unchanged, in this case bound to PDK1.

### 2.3 Tools

The LBS-*κ* language is supported by a web application which we describe in Subsection 2.3.1. We then demonstrate in Subsection 2.3.2 how the expressive power of LBS-*κ* can be increased through embedded scripts.

#### 2.3.1 A Web Application

The LBS-*κ* compiler translates LBS-*κ* programs to sets of Kappa rules, as defined by the above semantics. The compiler is implemented in the F# language. The parser implementation uses standard parser generator libraries (Lex and Yacc), and the code generator implementation follows a functional style naturally aligned with the definition of the LBS-*κ* semantics. Furthermore, the compiler is translated to JavaScript using the WebSharper kit, and is embedded in a web application; a screenshot is shown in Fig 3.

The web application provides a syntax-highlighting editor and a simulator for the flat Kappa models resulting from compilation of LBS-*κ* models. This simulator is implemented in F# and translated to JavaScript using WebSharper. We have adopted a simulation algorithm which uses just-in-time compilation to reactions, based on the framework introduced in [24]. Like the original simulation algorithm for Kappa [25], this avoids a compilation to a set of reactions which generally grows in proportion to the number of possible instantiations of the rules and which may even be infinite. Unlike the original simulator, our algorithm does not explicitly represent individual agents in the system and may for that reason yield improved performance for systems with large agent copy numbers. For systems with low agent copy numbers the original algorithm may perform better. Although the LBS-*κ* web application does not currently allow for the selection of simulators, introducing such an option is certainly possible and could be the subject of future work.

The web application is provided within a general framework, Bio Simulators, which allows for dynamic loading of simulator plugins via the web [26]. It is available at bsims.azurewebsites.net. The LBS-*κ* plugin can be selected through the menu shown at the top in the screenshot, available by clicking on the triangle next to the Bio Simulators title. A plugin for flat Kappa models based on the KaSim simulator from www.kappalanguage.org is also available.

#### 2.3.2 Embedded Scripts in LBS-*κ*

LBS-*κ* is a *domain specific language*: it is designed to naturally and succinctly capture processes in the specific domain of biochemistry. But at times more expressive power is needed, e.g. for generating large initial conditions as in the chemotactic switch ring case study. We here propose a simple solution using a general-purpose programming language as a scripting mechanism. This allows the domain specific features of LBS-*κ* to be used for the bulk of a model, while seamlessly calling upon the general-purpose language when needed for more complicated or ad hoc constructions. Our general purpose language of choice is F#, although any other language is in principle possible. We refer to segments of general purpose code as *scripts*, enclosed by **script** blocks within LBS-*κ*; scripts evaluate to strings which themselves are LBS-*κ* programs. The scripting feature is available for LBS generally, but we explain it here in terms of LBS-*κ*.

Below is an example of how 100 variants of an agent can be created and combined into a single non-deterministic agent S using a script. The resulting non-deterministic agent is subsequently used in a degradation rule (line 10) outside of the script, using the standard domain specific features of LBS-*κ*.

1 **script** {

2  let agentNames = seq {for i in 1.. 100 → “S” + string(i)} in

3  let newDefsLst =

4   Seq.map (fun s → “agent␣” + s + “␣=␣new{};”) agentNames in

5  let newDefsStr = String.concat “\n” newDefsLst in

6  let choiceStr = “agent␣S␣=␣” + (String.concat “␣or␣” agentNames) + “;” in

7  newDefsStr + “\n” + choiceStr

8 +

9 };

10

11 S →

Line 2 creates a sequence of a hundred strings representing agent names, S1 to S100. Lines 3-4 create a list of new agent definition strings, one for each agent name. Line 5 combines this list into a string. Line 6 creates a definition string for a non-deterministic agent S. The new agent definitions and non-deterministic agent definitions are finally combined and returned in line 7. The net effect of the script is, informally, to replace the script by the string resulting from evaluating the script. Hence the rule in line 8 is evaluated in an environment where the identifier S is bound to a non-deterministic species with a hundred variants, thus resulting in a hundred different degradation rules.

It is furthermore possible to define and reuse embedded scripts. This enables a second, general-purpose notion of modularity in LBS-*κ* through F# function definitions. The following example shows how the above F# code for generating a non-deterministic agent definition can be abstracted into an F# function called generate which is parameterised on an agent name and a number of variants; the function definition is then embedded within a **scriptdef** block, and subsequently invoked from within a separate **script** block.

1 **scriptdef** {

2  let generate name num =

3   let agentNames = seq {for i in 1.. num → name + string(i)} in

4   let newDefsLst =

5    Seq.map (fun s → “agent␣” + s + “␣=␣new{};”) agentNames in

6   let newDefsStr = String.concat “\n” newDefsLst in

7   let choiceStr =

8    “agent␣” + name + “␣=␣” + (String.concat “␣or␣” agentNames) in

9   newDefsStr + “\n” + choiceStr

10  in

11 };

12

13 **script**{

14  generate “S” 100

15 };

16

17 S →

We end by revisiting the chemotactic switch ring case study from Section 2.2.1. The full model includes an initial condition of a ring with 34 protomers. This initial condition can be defined with an embedded script as follows:

1 **script** {

2  “init␣” +

3   (

4    seq{

5    for i in 1.. 34 →

6     “P(f˜0, s, x!” + string(i%34) + “, y!” + string((i+1)%34) + “)”

7   }

8   |〉 String.concat “-”

9  ) + “␣1;”

10 }

Embedded scripts are only available in a Windows command line version of the LBS-*κ* tool [27] which also includes F# source code. The reason is that dynamic code generation in a web application is technically more involved and cannot be achieved purely using JavaScript. The formal foundations of the script language construct are outlined in Subsection 4.5.

## Discussion

We discuss related work in the first subsection before finally concluding.

### 3.1 Related Work

The languages Antimony and PySB mentioned in the introduction are closely related to LBS-*κ* in that they both address the problem of modularity in a rule-based setting. Being independently developed, the three languages have emerged with different sets of features and strengths. We compare each language to LBS-*κ* below; but common to both Antimony and PySB is that neither have language support for the LBS-*κ* features of agent expressions, agent subtyping in modules, compartment hierarchies, or non-determinism; further, neither has tools accessible directly through a web browser.

#### 3.1.1 Antimony

Antimony is designed to be a “modular human-readable, human-writeable model [28]. A key focus is on translation to SBML, and the language therefore directly supports SBML features such as events and inhibition/activation reaction types. Antimony also supports synthetic biology features such as DNA parts. A tool for editing models and compiling them to SBML is available for Windows and Mac OS.

As a consequence of its SBML focus, Antimony models express concrete reactions rather than Kappa-style rules as in LBS-*κ*. On a more fundamental level, the LBS framework is designed with formal foundations in mind, yielding the benefits outlined in the introduction.

#### 3.1.2 PySB

Both LBS-*κ* and Antimony can be considered domain specific languages. PySB, in contrast, can be described as an *embedded language*: models are written in the syntax of a general-purpose programming language, here Python, but in a style that fits the domain as closely as possible. This extends previous work on a Python-embedded language called Little b [29]. PySB provides seamless integration with tools for simulation and analysis of Kappa and BioNetGen models. A number of mature Python libraries for scientific computing are also readily available, which is a key strength of the embedded approach. Another strength is flexibility: since Python is a general-purpose programming language, there are no limitations on the models which can be generated.

Flexibility, however, also has a downside. Since anything is possible, bad things may also be possible. For example, a module may include a rule in which two reactant agents are bound using the binding label 1, and two additional reactant agents are parameters of the module. If these parameters are instantiated with agents bound using the same label 1, this would result in an error when the generated Kappa model is processed by the Kappa tools.

Another downside of embedded languages is their syntax, which, by definition, cannot escape the heritage of their general purpose host language. The following is an example of a PySB catalysis module adapted from the supplementary material of [18], with rule labels removed for clarity:

1 def catalyze(enz, e_site, sub, s_site, prod, klist):

2  kf, kr, kc = klist

3  Rule(enz({e_site:None}) + sub({s_site:None}) 〈〉

4  enz({e_site:1}) % sub({s_site: 1}), kf, kr)

5  Rule(enz({e_site:1}) % sub({s_site: 1}) 〉〉

6  enz({e_site:None}) + prod({s_site:None}), kc)

While the meaning of this module is fairly clear due to the use of Python operator overloading for e.g. +, % (complexes), 〉 〉 (reactions) and 〈 〉 (reversible reactions), some syntactic overhead is still needed; for example, the Rule identifier must be used. Note also how site parameters are independent from the corresponding agent parameter, so in order to use the module it may be necessary to inspect the module body to determine which site belongs to which agent. Finally, site values are Python primitives, meaning that a single site cannot have both internal state and binding state; it may hence be necessary to artificially separate one site into two distinctly named sites. We note that although these points do apply to PySB, they are not necessarily general points against embedded languages: embeddings into other languages with different trade-offs may be possible.

S5 Listing shows a more comprehensive PySB example, namely that of a MAPK cascade, thereby affording a direct comparison with the corresponding LBS-*κ* MAPK cascade model. Both models define a cycle module which in turn contains sub-modules for phosphorylation and dephosphorylation, and both instantiate the cycle module five times. As with the example above one sees that the PySB model is significantly more verbose and that site parameters are listed independently from their corresponding agents. One also sees how separate sites are needed for binding and modification.

### 3.2 Conclusion

We have introduced the LBS-*κ* language for writing high-level, modular rule-based models, and we have illustrated its use via a number of small examples and three larger case studies. The first two case studies are characterised by repeated structure which can be expressed naturally using LBS-*κ* modularity. The third, and largest, case study, a model of insulin signalling exhibits no obvious large scale modularity, but the model nevertheless benefits from the use of modules for common rules and triplets of rules, and from other language constructs such as agent expressions and non-determinism.

The LBS-*κ* language has been formally defined, and a compiler from LBS-*κ* models to flat Kappa models has been implemented and made available through a web application. We have contrasted two approaches to language-based modelling, namely through DSLs and embedded languages, and we have demonstrated one way of combining both approaches through the use of F# scripts directly within LBS-*κ*. We view this discussion as being of wider relevance to modelling language design, as the notion of embedded scripts is not LBS-*κ* specific.

As regards future work, the question of whether *large* multi-instance modules of the kind discussed here really exist in natural, evolved systems remains open. But in synthetic biology, where novel systems are engineered for useful purposes, abstraction through modularity is likely to become an important means for coping with complexity. Modular DSLs targeted to the synthetic biology domain may hence be of increasing interest. With respect to language design, one promising direction is to extend Kappa sites with a notion of *colour* as in coloured Petri nets [13, 30], allowing more elaborate conditions on internal state and changes thereof during rule application.

## Methods

We now turn to the formal definition of LBS-*κ*. The LBS framework is parameterised on two structures. The first is a *syntactic* parameter which specifies a syntax for agent site expressions. The second is a *semantic* parameter which specifies the target semantical objects and relevant operations on these. In the case of LBS-*κ*, the target semantical objects are flat Kappa programs. The semantic parameter generally depends on the syntactic one: only agents which have a notion of binding in their site expressions can be translated to Kappa.

The LBS framework [14, 15] provides a general *syntax*, for example that of module definitions and invocations, that is independent of any particular choice of agent site expressions. For the sake of completeness, we reproduce selected parts of this general syntax in Section 4.2, where we also define the syntax of agent site expressions which is specific to the LBS-*κ* instantiation.

The LBS framework also provides a general *semantics*, specifying how language constructs such as module definitions and invocations are translated independently of any particular target semantics such as Kappa. We do not reproduce the full general semantics here, but refer instead to [14, 15]. We do however outline the general semantics framework in Section 4.3 in order to define the structure of the LBS semantic parameter. We then proceed to define the Kappa semantic parameter in Section 4.4, thereby completing the formal definition of the instantiation of LBS to LBS-*κ*.

### 4.1 Notation

We let ℝ denote the set of *real numbers* ranged over by *r* and we let ℕ denote the set of *natural numbers* ranged over by *k*. We write $\underline{x}$ for *lists*, $\underline{x}.i$ for the **i*th element* (starting from 1) of a list, $\left|\underline{x}\right|$ for the *length* of a list and *ɛ* for the *empty* list. The *concatenation* of lists $\underline{x}$ and $\underline{y}$ is written $\underline{x}\;\underline{y}$, and the *prefix* of an element *a* to a list $\underline{x}$ is written $a\underline{x}$.

Given a set *X* we write X* for the *Kleene closure* of *X*. We write {*x*_*i*_}_*i*∈*I*_ for a finite *indexed set* and omit *I* and/or *i* and write {*x*_*i*_}_*I*_, {*x*_*i*_} or {*x*} when they are understood from the context. Partial finite *functions* *f*:*X* ↪_fin_ *Y* are denoted by finite indexed sets of pairs {*x*_*i*_ ↦ *y*_*i*_} where *f*(*x*_*i*_) = *y*_*i*_. The *domain of definition* and *image* of a function *f* are denoted by *dom*(*f*) and *im*(*f*), respectively. We specify the type of a partial function *f* by writing *f*(*x*) = *y* where *x* and *y* are given variables ranging over two sets; the sets are then understood to form the domain and image of *f*.

We write $e\;\overset{\Delta }{≃}\;e^{\prime }$ for definitions where the expression *e* equals the expression *e* ^′^ if *e* ^′^ is defined, and where *e* is undefined otherwise. When a notion of well-typedness applies to *e* ^′^, we furthermore write $e\;{\overset{\Delta }{≃}}_{\text{t}}\;e^{\prime }$ for definitions where *e* equals *e* ^′^ if *e* ^′^ is defined and well-typed, and where *e* is undefined otherwise.

For easy reference some of the key symbols appearing in the syntax and semantics definitions are given in Table 3.

**Table 3:** The abstract syntax for LBS-*κ* site types and expressions.

*ρ*	∷=	**binding**(*V*)	Site types
*e*_s_	∷=	(*i*, *l*)	Site expressions
*i*	∷=		Internal state
	∣	*v*	Internal state value
	∣	?	Wild card
	∣	*ɛ*	Identity
*l*	∷=		Link
	∣	?	Free or bound
	∣	_	Bound to something
	∣	⌘	Free
	∣	(*k*, *b*)	Restricted link label
	∣	*ɛ*	Identity

### 4.2 The Abstract Syntax of LBS-*κ*

The *abstract* syntax of LBS-*κ* forms the foundation for its subsequent semantics. A formal definition of the *concrete* syntax of LBS-*κ* and its mapping into the abstract syntax are omitted. Both can be deduced without surprises from the abstract syntax and the examples in the previous section.

#### 4.2.1 Agent Expressions

The abstract syntax for agent expressions is given in Table 4, where *n*_a_ ranges over a finite set of *agent names*, *n*_s_ ranges over a finite set of *site names*, *id*_a_ ranges over a finite set of *agent identifiers* and *id*_c_ ranges over a finite set of *compartment identifiers* (note that in [14, 15] we used *n*_*s*_ for species/agent names and *n*_*m*_ for site names; we have adopted a different notation here to better reflect the usual Kappa terminology). Agent names identify atomic agents independently of any sites, while agent identifiers refer to possibly complex expressions including both the names and site states of atomic agents in the complex. Table 4 specifically omits the definition of site types *ρ* and site expressions *e*_s_ since these are parameters of the syntax. They are defined separately in the following. Note that several abbreviations can be defined in terms of the basic abstract syntax in Table 4; some of these abbreviations are mentioned below.

**Table 4:** The abstract syntax for LBS-*κ* programs.

*P*	∷=		Program
	∣	$\underline{e_{\text{a}}}\;\to^{e_{\text{r}}}\;\underline{e_{\text{a}}^{\prime }}$	Rule
	∣	*P* ∣ *P* ^′^	Parallel composition
	∣	*id*_c_[*P*]	Located program
	∣	*D*; *P*	Definition
	∣	${\text{id}}_{\text{m}}(\underline{{\text{id}}_{\text{c}}};\underline{e_{\text{a}}};\underline{e_{\text{r}}})$	Module invocation
	∣	**init** *e*_a_ *n*	Initial population
	∣	**nil**	Empty program
*e*_r_	∷=		Rate expression
	∣	*r*	Constant
	∣	*id*_r_	Rate identifier

New agents are created by specifying a name and a type consisting of a partial finite function from site names to site types; as shown in Section 2.1.2, the agent name can be left out as an abbreviation in definitions in which case the identifier, to which the new agent is assigned, is assumed as the name. Location allows agents to span compartments within rules. For this reason updates generally include compartments in addition to an agent name and a site update expression; updates on atomic agents may however omit the agent name as an abbreviation. Annotations are used when agents are passed as actual parameters in module instantiations: they specify how located atomic agents and sites should be mapped to the located atomic agents and sites in the corresponding formal agent parameter which also includes an annotation. An informal explanation of this mapping, but without location, is given in Section 2.1.4. Examples using the choice operator for writing non-deterministic rules which expand to many concrete rules are given in Section 2.1.6. The composition and identifier expressions are straightforward and have often been used in the above examples.

Let 𝒱 be a given finite set of *internal state values*. Binding site types and binding expressions specific to LBS-*κ* are then defined by the abstract syntax in Table 5, where *V* ⊂ 𝒱 ranges over sets of internal state values, *v* ∈ 𝒱 ranges over individual internal state values and *b* ∈ {0, 1}* is used to create *namespaces* for confining link labels within modules.

**Table 5:** The abstract syntax for definitions.

*D*	∷=		Definition
	∣	*id*_a_ = *e*_a_	Agent
	∣	*id*_c_ = **new comp**	New top-level compartment
	∣	*id*_c_ = **new comp inside** *id*_c_	New nested compartment
	∣	*id*_r_ = *e*_r_	Rate
	∣	${\text{id}}_{\text{m}}(\underline{{\text{id}}_{\text{c}}};\underline{{\text{id}}_{\text{a}}:\xi };\underline{{\text{id}}_{\text{r}}})=P$	Module

A site type simply consists of a set of allowed internal state values. A site expression is a pair consisting of an internal state *i* and a link *l*. An internal state can be a value *v*, such as “phosphorylated” or “unphosphorylated”; it can be a wild card indicating “any” value; or it can be the identity *ɛ* for use in updates when the internal state should remain unaffected. A link can be one of two kinds of wild cards, with the more permissive being “either free or bound” and the more restricted being “bound to something”; a link can also be free, i.e. unbound; it can be bound by some restricted link label which is given by a label *k* together with a namespace index *b* ∈ {0, 1}*; or it can be the identity *ɛ* for use in updates when the link state should remain unaffected. The internal state may be omitted from a site expression as an abbreviation, in which case the wild card, ?, is assumed, and the link may be omitted as an abbreviation in which case the free link, ο, is assumed. If both are omitted, the pair (?, ο) is assumed.

#### 4.2.2 Programs

The abstract syntax of programs is given in Table 6, where *n* ∈ ℕ, *id*_m_ ranges over the set of *module identifiers*, *id*_r_ ranges over the set of *rate identifiers* and *id*_c_ again ranges over the set of compartment identifiers. Definitions, ranged over by *D*, are defined below. The grammar closely matches the syntax used in the examples and should be self-explanatory, perhaps with the exception of initial populations and nil programs: the former are specified using the **init** keyword and can occur anywhere in a program, and the latter acts as the identity element under parallel composition: this can be useful for advanced constructions.

**Table 6:** The abstract syntax for Kappa rules.

*x*	∷=	$\underline{a}\to^{r}\underline{a^{\prime }}$	Kappa rule
*a*	∷=	$\left(n_{\text{a}},\underline{s}\right)$	Agent
*s*	∷=	(*n*_s_, *i* ^−^, *l* ^−^)	Site
*i* ^−^	∷=		Internal state
	∣	*v*	Internal state value
	∣	?	Wild card
*l* ^−^	∷=		Link
	∣	?	Free or bound
	∣	_	Bound to something
	∣	ο	Free
	∣	*k*	Link label

There are some evident abbreviations. For example, a reversible rule corresponds to the parallel composition of the rules for each direction, and the definition of an agent expression within a rule using the! as! keyword can be expressed using a standard agent definition prior to the rule as detailed in [14, 15].

#### 4.2.3 Definitions

The abstract syntax for definitions is shown in Table 5. Note that compartments, like agents, are defined using the **new** keyword, and unless a compartment is used at the top level (the first case), a parent must be specified (the second case). Formal agent parameters in module definitions have annotations *ξ* as defined in the abstract syntax for agent expressions and as demonstrated through examples in Section 2.1.4. Together with the corresponding annotation of actual agent parameters which are included in the abstract syntax for agent expressions, this is sufficient to construct a mapping that allows use of the agents inside the module body after invocation; we refer to Section 5.2.1 and Section 5.4 of [14] for the full technical details.

### 4.3 The General Semantics Framework

The general semantics framework of LBS is independent of any particular target semantics such as Kappa. The framework hence defines, in general terms, the meaning of e.g. agent updates, location, module definitions and module invocations. As a result, the general semantics need not be redefined for each concrete semantics under study: all that is needed is the definition of the functions on which the general semantics is parameterised. We hence refer to [14, 15] for the definition of the general semantics, which also includes instantiations to concrete semantics for ordinary differential equations, continuous time Markov chains and Petri nets; in these cases agents in the abstract syntax have no binding structure.

The general semantics evaluates individual rules to *normal form rules* *R* as defined by the following grammar:

$$\begin{array}{c} \begin{array}{ccc} R & ::= & \;\;\underline{v_{\mathrm{na}}}\;\Rightarrow^{r}\;\underline{v_{\mathrm{na}}^{'}}\\ v_{\mathrm{na}} & ::= & \;\;\underline{\underline{n_{c}}\left[n_{a},\alpha_{\sigma }\right]}\\ \alpha_{\sigma } & ::= & \;\;\{n_{s}\mapsto \left(\rho ,e_{s}\right)\} \end{array} \end{array}$$

A ground normal form rule is like a rule but with rate expressions evaluated to rate constants and agent expressions evaluated to *normal form agent values*, *v*_na_; normal form agent values are lists of triples with a comparment list for identifying location, an agent name, and a *typed site assignment* *α*_*σ*_ which to each site assigns a type *ρ* and a site expression *e*_s_. Note that the site type and expressions constitute the parameters of the general syntax. Note also that the general semantics includes an additional *ground* normal form of rules and agents, but that distinction is not needed in the LBS-*κ* context where site expressions have no variables.

In order to produce normal form rules from a model the general semantics is parameterised on the following relation and functions pertaining to site types *ρ* and site expressions *e*_s_:

- A *typing relation* of the form *e*_s_ : *ρ* giving types to site expressions. This is used for determining well-typedness of agent expressions.
- A *default expression function* of the form *default*(*ρ*) = *e*_s_ giving default expressions to site types. This is used to assign site expressions to unassigned sites in agent expressions.
- An *update function* of the form $e_{\text{s}}\langle e_{\text{s}}^{\prime }\rangle =e_{\text{s}}^{\prime \prime }$ for updating one site expression with another. This is used in the semantics of agent update expressions.
- A *seal function* of the form $\text{seal}(e_{\text{s}},b)=e_{\text{s}}^{\prime }$ for confining names in site expressions to a *namespace* given by a binary string *b* ∈ {0, 1}*. This is used to avoid capture of free names (link labels in the Kappa context) when agent expressions are passed as module parameters.

In order to produce a concrete semantical object such as a flat Kappa model from an LBS-*κ* model, the general semantics framework is further parameterised, on a *concrete semantics* structure (*S*, ∣_*S*_, **0**_*S*_, *R*_*S*_, *I*_*S*_) consisting of:

- A set *S* of *semantical objects* ranged over by 𝒪.
- A partial binary *composition function* ∣_*S*_ on semantical objects.
- A distinguished *nil semantical object* **0**_*S*_ ∈ *S*.
- A partial *rule assignment function* of the form *R*_*S*_(*R*, *b*) = 𝒪 assigning a semantical object to a given normal form rule *R* named *b*.
- A partial *initial condition assignment function* of the form *I*_*S*_(*v*_na_, *k*) = 𝒪 assigning a semantical object to an initial population *k* of a normal form agent value *v*_na_.

### 4.4 The Concrete Kappa Semantics

We define the relations and functions pertaining to site types and expressions in the Kappa instantiation as follows, where IV(*e*_s_) denotes the (singleton or empty) set of internal state values in the site expression *e*_s_:

- *e*_s_: **binding**(V) for all *e*_s_ and *V* with IV(*e*_s_) ⊆ *V*
- *default*(**binding**(V)) $\overset{\Delta }{≃}$ (?, ?)
- (*i*, *l*)⟨(*i* ^′^, *l* ^′^)⟩ $\overset{\Delta }{≃}$ *i*⟨*i* ^′^⟩, *l*⟨*l* ^′^⟩) where
    – $i\langle i^{\prime }\rangle \overset{\Delta }{≃}\left\{ \begin{array}{cc} i & \text{if}\;i^{\prime }=ɛ\\ i^{\prime } & \text{otherwise} \end{array}\right.$
    – $l\langle l^{\prime }\rangle \overset{\Delta }{≃}\left\{ \begin{array}{cc} l & \text{if}\;l^{\prime }=ɛ\\ l^{\prime } & \text{otherwise} \end{array}\right.$
- $\text{seal}((i,l),b)\overset{\Delta }{≃}\left\{ \begin{array}{cc} \left(i,(k,bb^{\prime })\right) & \text{if}\;l=(k,b^{\prime })\\ \left(i,l\right) & \text{otherwise}\; \end{array}\right.$

The typing relation asserts that a binding expression can only use internal state values declared by its type. The default expression for unspecified modification site types has a wild card internal state and link, which reflects the use of unspecified sites in Kappa. The update function overwrites any internal state or links in all cases except when the identity is used for updating. Finally, the seal function simply updates the namespace of any link labels by concatenating the given binary string to the binary string already present.

Next we define the concrete semantics structure for Kappa. The abstract syntax for Kappa rules is given in Table 6 where, as before, *n*_a_ ranges over the set of agent names, *n*_s_ ranges over the set of site names, *r* ∈ ℝ and *k* ∈ ℕ. Furthermore, $v\in 𝒱\cup \underline{N_{\text{c}}}$ ranges over a given set of internal state values as in LBS-*κ*, but also over the set of compartment name lists; the latter is needed in order to encode LBS-*κ* compartments in Kappa. The abstract syntax for Kappa rules follows that of the literature (see e.g. [31]), but is adapted notationally for our purposes.

A Kappa rule consists of a list of reactant agents and a list of product agents, and the arrow is labelled with a rate constant. A Kappa agent is similar to an LBS-*κ* agent, with the exception that, following the practices of Kappa, site expressions are lists rather than functions and the identity *ɛ* is omitted from internal and link states.

A Kappa program *K* is then a pair (*X*, *I*) where *X* is a set {*x*_*i*_} of Kappa rules and *I* is a list $\underline{\underline{a}}$ of complexes (which in turn are lists of agents) representing the initial conditions for simulation. An agent is well-typed if each of its site names occurs exactly once; a list of agents is well-typed if all the agents are well-typed and each link label occurs exactly twice; a rule is well-typed if its two lists of agents are well-typed; initial conditions are well-typed if all agents are *ground*, i.e. they contain no wild cards; and finally, a Kappa program is well-typed if all its rules and its initial conditions are well-typed. We denote by 𝒦 the set of all well-typed Kappa programs.

We are now in a position to define two elements of the concrete semantics structure for Kappa, namely the the parallel composition and the nil object:

- *K*_1_ ∣_𝒦_ *K*_2_ $\overset{\Delta }{≃}$ where
    – *X*_*K*_ ${\overset{\Delta }{≃}}_{\text{t}}$ *X*_*K*1_∪*X*_*K*_2__
    – *I*_*K*_ ${\overset{\Delta }{≃}}_{\text{t}}$ *I*_*K*1_ *I*_*K*_2__
- **0**_𝒦_ $\overset{\Delta }{≃}$ (∅, *ɛ*)

We define the remaining elements below.

The sites of an LBS-*κ* agent are represented by finite functions rather than lists as in Kappa. The translation to Kappa must therefore “linearise” these functions, for which we assume a linear ordering, ≤, on site names.

It must also convert restricted link labels to natural-number link labels, for which we assume an injective function of the form *enc*(*k*, *b*) = *k* ^′^; a definition could e.g. be based on a Gödel numbering. Given an LBS-*κ* typed site assignment *α*_*σ*_ = {*n*_s__*j*_ ↦ (*ρ*, (*i*, *l*))_*j*_} we then define *kap*_s_(*α*_*σ*_) to be the list with the element (*n*_s__*j*_, *i*_*j*_, *enc*(*l*_*j*_)) at index |*S*| where

$$\begin{array}{c} S\overset{\Delta }{≃}n_{s}\in \mathrm{dom}\left(\alpha_{\sigma }\right)|n_{s}\leq {n_{s}}_{j}\} \end{array}$$

We here assume *enc* extended to LBS-*κ* links in an evident manner. Note that site types are not needed for the Kappa translation, and so are discarded.

The translation of an LBS-*κ* agent to a Kappa agent simply translates the agent’s sites and adds an additional site with an internal state representing the enclosing compartments. For the latter we assume a distinguished site name, *comp*. This ensures that agents in different compartments are distinguished.

We now define a *kappa translation function* of the form $\text{kap}(v_{\text{na}})=\underline{a}$ for translating normal form agent values to lists of Kappa agents as follows:

$$\begin{array}{c} \mathrm{kap}\left(\underline{n_{c}}\left[n_{a},\alpha_{\sigma }\right]\right)\overset{\Delta }{≃}\;(n_{a},{\mathrm{kap}}_{s}\left(\alpha_{\sigma }\right)\left(\mathrm{comp},\underline{n_{c}},ο\right)) \end{array}$$

where ${\text{kap}}_{\text{s}}(\alpha_{\sigma })(\text{comp},\underline{n_{\text{c}}},ο)$ following our notational conventions is the postfixing of the triple $\left(\text{comp},\underline{n_{\text{c}}},ο\right)$ to the list *kap*_s_(*α*_*σ*_). LBS-*κ* rules are translated into Kappa rules by applying the above function to each normal form agent value and flattening the lists representing reactants and products. In the following we therefore assume a given function *flatten* for flattening lists, and also a given function of the form $k\times \underline{a}=\underline{\underline{a^{\prime }}}$ which generates a list $\underline{\underline{a^{\prime }}}$ with *k* copies of the agent list $\underline{a}$.

The concrete semantics for LBS-*κ* in terms of Kappa is then given by the tuple (𝒦, ∣_𝒦_, **0**_𝒦_, *G*_𝒦_, *I*_𝒦_) where the first three elements are defined above and the last two elements are defined as follows:

- $G_{𝒦}(\underline{v_{\text{na}}}\;\Rightarrow^{r}\;\underline{v_{\text{na}}^{\prime }},b){\overset{\Delta }{≃}}_{\text{t}}$ *K* where
    – ${\text{X}}_{\text{K}}\;{\overset{\Delta }{≃}}_{\text{t}}\;\left\{\text{flatten}(\underline{\text{kap}(v_{\text{na}})}\to^{r}\text{flatten}(\underline{\text{kap}(v_{\text{na}}^{\prime })})\right\}$
    – *I*_*K*_ $\overset{\Delta }{≃}$ *ε*
- *I*_𝒦_(*v*_na_, *k*) ${\overset{\Delta }{≃}}_{\text{t}}$ *K* where
    – *X*_*K*_ $\overset{\Delta }{≃}$ ∅
    – *I*_*K*_ ${\overset{\Delta }{≃}}_{\text{t}}$ *k* ×*kap*(*v*_na_)

Note that the rule assignment function is only defined if the resulting Kappa rule is well-typed, and the initial conditions assignment function is only defined if the resulting agent list is well-typed. Note too that well-typedness of agent expressions with respect to link labels is only determined by the general semantics when the concrete semantics is applied to rules. A dedicated type system would be needed to determine well-typedness earlier, e.g. at species definition time.

The translation can be adapted with minor modifications to target BioNetGen. There, agents in rules are separated by connectivity as in LBS-*κ*, meaning that rules need not be flattened during translation.

### 4.5 Scripts

The scripting feature, demonstrated in Subsection 2.3.2, is not defined in the general syntax and semantics for LBS presented in [14, 15]. We now outline how this can be done. One extends the syntax of LBS-*κ* by two more productions for programs:

$$\begin{array}{c} \begin{array}{cccc} P & ::= & & \text{PROGRAM}\\ & \;|\;\; & \mathrm{script}\;F_{1}\;;\;P & \text{SCRIPT}\\ & \;|\;\; & \mathrm{scriptdef}\;F_{2}\;;\;P & \text{SCRIPT}\;\text{DEFINITION} \end{array} \end{array}$$

where *F*_1_ and *F*_2_ are F# programs satisfying the following conditions:

- *F*_1_ has type *string*, and its string value must be an LBS-*κ* program with a *hole* at the end. Since the hole is always at the end, it has no explicit syntax, and in practice is preceded by either the “;“ or “|“ operator as shown in the examples.
- *F*_2_ is a top-level expression containing definitions.

Semantically, the contents of **scriptdef** blocks are collected into a script environment during compilation of an LBS-*κ* model; the environment is simply a string, consisting of the concatenation of any script definitions in scope at a given point during compilation. A script of the form **script** *F*_1_; *P* is evaluated by first appending *F*_1_ to the current script environment and then compiling the resulting F# code using the standard F# compiler (through the F# CodeDom library). The compiled code is then executed to obtain an LBS-*κ* program string with a trailing hole. This string is parsed using the LBS-*κ* parser extended to accommodate holes, resulting in an abstract syntax tree (AST) for the script. The AST for *P*, i.e. the sequential LBS-*κ* program, is then inserted into the hole of the first AST, and the compiler recurses on the resulting AST, keeping the same script environment.

## Supporting Information

S1 Listing A flat Kappa model of the chemotaxis switch ring. This is part of a larger model by Vincent Danos, reproduced with permission from the author.
(PDF)

S2 Listing A flat Kappa MAPK cascade model adapted from [21]. Reproduced with permission from the author but with some sites renamed and the rules reorganised to better reflect the underlying modular structure.
(PDF)

S3 Listing A flat Kappa model of insulin signalling by Isha Antani and Gordon Webster. The model is reproduced with permission from the second author, but with rule labels and comments removed.
(PDF)

S4 Listing A modular LBS-*κ* insulin signalling model. (PDF)

S5 Listing An example PySB model of a MAPK cascade. Included for comparison with the LBS-*κ* MAPK cascade model. Lines 1-15 define the cycle module. Lines 17-25 define the agents, here called *monomers*. Lines 27-31 contain the module instantiations. Each of these three blocks have similar blocks in the corresponding LBS-*κ* model. Because PySB distinguishes between sites for binding and sites with internal states, some sites are separated into two in the PySB model; we use the “b”-prefix for sites which are used for binding, and the “m”-prefix for sites which have modification state. The syntax of PySB is otherwise inherited from Python, a detailed description of which is outside the scope of this comparison. We note that, although we have attempted to construct a model which is equivalent to the LBS-*κ* model, we have not been able to verify that the the models do indeed translate to equivalent flat models.
(PDF)
