# Supplementary material for: A High-Level Language for Rule-Based Modelling
Source: PLoS One. 2015 Jun 4;10(6):e0114296. doi: 10.1371/journal.pone.0114296 (PMC4456403; doi:10.1371/journal.pone.0114296)
Supplement: S1 Listing — This is part of a larger model by Vincent Danos, reproduced with permission from the author. (PDF) [file pone.0114296.s001.pdf]

Listing S1: A flat Kappa model of the chemotaxis switch ring. This is part of a larger model by Vincent Danos, reproduced with permission from the author.

```
## 8 flips (aka conformational change)
### 4 P flips without CheY - note that P(f~0) is favoured 2/1
'flip 000'  P(f~0,y!1),P(x!1,f~0,y!2,s),P(x!2,f~0) → P(f~0,y!1),P(x!1,f~1,y!2,s),P(x!2,f~0)@1
'bflip 000' P(f~0,y!1),P(x!1,f~1,y!2,s),P(x!2,f~0) → P(f~0,y!1),P(x!1,f~0,y!2,s),P(x!2,f~0)@200

'flip 100'  P(f~1,y!1),P(x!1,f~0,y!2,s),P(x!2,f~0) → P(f~1,y!1),P(x!1,f~1,y!2,s),P(x!2,f~0)@1
'bflip 100' P(f~1,y!1),P(x!1,f~1,y!2,s),P(x!2,f~0) → P(f~1,y!1),P(x!1,f~0,y!2,s),P(x!2,f~0)@2

'flip 001'  P(f~0,y!1),P(x!1,f~0,y!2,s),P(x!2,f~1) → P(f~0,y!1),P(x!1,f~1,y!2,s),P(x!2,f~1)@1
'bflip 001' P(f~0,y!1),P(x!1,f~1,y!2,s),P(x!2,f~1) → P(f~0,y!1),P(x!1,f~0,y!2,s),P(x!2,f~1)@2

'flip 101'  P(f~1,y!1),P(x!1,f~0,y!2,s),P(x!2,f~1) → P(f~1,y!1),P(x!1,f~1,y!2,s),P(x!2,f~1)@100
'bflip 101' P(f~1,y!1),P(x!1,f~1,y!2,s),P(x!2,f~1) → P(f~1,y!1),P(x!1,f~0,y!2,s),P(x!2,f~1)@2

### 4 P flips with CheY - note that all forwards are multiplied by 10
'flip 000b'  P(f~0,y!1),P(x!1,f~0,y!2,s!_),P(x!2,f~0) → P(f~0,y!1),P(x!1,f~1,y!2,s!_),P(x!2,f~0)@10
'bflip 000b' P(f~0,y!1),P(x!1,f~1,y!2,s!_),P(x!2,f~0) → P(f~0,y!1),P(x!1,f~0,y!2,s!_),P(x!2,f~0)@200

'flip 100b'  P(f~1,y!1),P(x!1,f~0,y!2,s!_),P(x!2,f~0) → P(f~1,y!1),P(x!1,f~1,y!2,s!_),P(x!2,f~0)@10
'bflip 100b' P(f~1,y!1),P(x!1,f~1,y!2,s!_),P(x!2,f~0) → P(f~1,y!1),P(x!1,f~0,y!2,s!_),P(x!2,f~0)@2

'flip 001b'  P(f~0,y!1),P(x!1,f~0,y!2,s!_),P(x!2,f~1) → P(f~0,y!1),P(x!1,f~1,y!2,s!_),P(x!2,f~1)@10
'bflip 001b' P(f~0,y!1),P(x!1,f~1,y!2,s!_),P(x!2,f~1) → P(f~0,y!1),P(x!1,f~0,y!2,s!_),P(x!2,f~1)@2

'flip 101b'  P(f~1,y!1),P(x!1,f~0,y!2,s!_),P(x!2,f~1) → P(f~1,y!1),P(x!1,f~1,y!2,s!_),P(x!2,f~1)@1000
'bflip 101b' P(f~1,y!1),P(x!1,f~1,y!2,s!_),P(x!2,f~1) → P(f~1,y!1),P(x!1,f~0,y!2,s!_),P(x!2,f~1)@2
```
