# Supplementary material for: A High-Level Language for Rule-Based Modelling
Source: PLoS One. 2015 Jun 4;10(6):e0114296. doi: 10.1371/journal.pone.0114296 (PMC4456403; doi:10.1371/journal.pone.0114296)
Supplement: S2 Listing — Reproduced with permission from the author but with some sites renamed and the rules reorganised to better reflect the underlying modular structure. (PDF) [file pone.0114296.s002.pdf]

Listing S2: A flat Kappa MAPK cascade model adapted from [1]. Reproduced with permission from the author but with some sites renamed and the rules reorganised to better reflect the underlying modular structure.

```
Ras(n~gtp),Raf(n~u) → Ras(n~gtp!1),Raf(n~u!1)
Ras(n~gtp!1),Raf(n~u!1) → Ras(n~gtp!1),Raf(n~p!1)
Ras(n~gtp!1),Raf(n!1) → Ras(n~gtp),Raf(n)
PP2A1(n),Raf(n~p) → PP2A1(n!1),Raf(n~p!1)
PP2A1(n!1),Raf(n~p!1) → PP2A1(n!1),Raf(n~u!1)
PP2A1(n!1),Raf(n!1) → PP2A1(n),Raf(n)

Raf(n~p),MEK(S222~u) → Raf(n~p!1),MEK(S222~u!1)
Raf(n~p!1),MEK(S222~u!1) → Raf(n~p!1),MEK(S222~p!1)
Raf(n~p!1),MEK(S222!1) → Raf(n~p),MEK(S222)
PP2A2(n),MEK(S222~p) → PP2A2(n!1),MEK(S222~p!1)
PP2A2(n!1),MEK(S222~p!1) → PP2A2(n!1),MEK(S222~u!1)
PP2A2(n!1),MEK(S222!1) → PP2A2(n),MEK(S222)

Raf(n~p),MEK(S218~u) → Raf(n~p!1),MEK(S218~u!1)
Raf(n~p!1),MEK(S218~u!1) → Raf(n~p!1),MEK(S218~p!1)
Raf(n~p!1),MEK(S218!1) → Raf(n~p),MEK(S218)
PP2A2(n),MEK(S218~p) → PP2A2(n!1),MEK(S218~p!1)
PP2A2(n!1),MEK(S218~p!1) → PP2A2(n!1),MEK(S218~u!1)
PP2A2(n!1),MEK(S218!1) → PP2A2(n),MEK(S218)

MEK(n,S218~p,S222~p),ERK(T185~u) → MEK(n!1,S218~p,S222~p),ERK(T185~u!1)
MEK(n!1,S218~p,S222~p),ERK(T185~u!1) → MEK(n!1,S218~p,S222~p),ERK(T185~p!1)
MEK(n!1,S218~p,S222~p),ERK(T185!1) → MEK(n,S218~p,S222~p),ERK(T185)
MKP3(n),ERK(T185~p) → MKP3(n!1),ERK(T185~p!1)
MKP3(n!1),ERK(T185~p!1) → MKP3(n!1),ERK(T185~u!1)
MKP3(n!1),ERK(T185!1) → MKP3(n),ERK(T185)

MEK(n,S218~p,S222~p),ERK(Y187~u) → MEK(n!1,S218~p,S222~p),ERK(Y187~u!1)
MEK(n!1,S218~p,S222~p),ERK(Y187~u!1) → MEK(n!1,S218~p,S222~p),ERK(Y187~p!1)
MEK(n!1,S218~p,S222~p),ERK(Y187!1) → MEK(n,S218~p,S222~p),ERK(Y187)
MKP3(n),ERK(Y187~p) → MKP3(n!1),ERK(Y187~p!1)
MKP3(n!1),ERK(Y187~p!1) → MKP3(n!1),ERK(Y187~u!1)
MKP3(n!1),ERK(Y187!1) → MKP3(n),ERK(Y187)
```

References

[1] Vincent Danos. Agile modelling of cellular signalling. *Electr. Notes Theor. Comput. Sci.*, 229(4):3–10, 2009.
