# Supplementary material for: A High-Level Language for Rule-Based Modelling
Source: PLoS One. 2015 Jun 4;10(6):e0114296. doi: 10.1371/journal.pone.0114296 (PMC4456403; doi:10.1371/journal.pone.0114296)
Supplement: S3 Listing — The model is reproduced with permission from the second author, but with rule labels and comments removed. (PDF) [file pone.0114296.s003.pdf]

Listing S3: A flat Kappa model of insulin signalling by Isha Antani and Gordon Webster. The model is reproduced with permission from the second author, but with rule labels and comments removed.

```
IGF1R(Y1131~u,alpha) → IGF1R(Y1131~p,alpha)
PI3K-canon(subs!1), PIP-canon(three!1) →
  PI3K-canon(subs), PIP-canon(three)
GEF(gef!1), Rab10(g!1) → GEF(gef), Rab10(g)
PTP1B(receptor!1), IGF1R(Y1131~p,Y1136~p,Y1135~p,NPXY~p,ptp!1) →
  PTP1B(receptor!1), IGF1R(Y1131~u,Y1136~u,Y1135~u,NPXY~u,ptp!1)
PDK1(PH), PIP-canon(three~p) → PDK1(PH!1), PIP-canon(three~p!1)
Akt-canon(foxo4!1), FoxO4(T28~u,akt!1) →
  Akt-canon(foxo4!1), FoxO4(T28~p,akt!1)
Akt-canon(foxo4!1), FoxO4(S193~u,akt!1) →
  Akt-canon(foxo4!1), FoxO4(S193~p,akt!1)
IGF1R(alpha,NPXY~u) → IGF1R(alpha,NPXY~p)
GSK3b(S9~u,a), Akt-canon(T308~p,subs,S473~p) →
  GSK3b(S9~u,a!1), Akt-canon(T308~p,subs!1,S473~p)
FoxO4(S193~p,T28~p,p1433), P1433(dum~off,foxo4) →
  FoxO4(S193~p,T28~p,p1433!1), P1433(dum~on,foxo4!1)
mTOR(sin), SIN1(m) → mTOR(sin!1), SIN1(m!1)
PTP1B(receptor), IGF1R(Y1131~p,Y1136~p,Y1135~p,NPXY~p,ptp) →
  PTP1B(receptor!1), IGF1R(Y1131~p,Y1136~p,Y1135~p,NPXY~p,ptp!1)
FoxO4(p1433!1), P1433(dum~on,foxo4!1) → FoxO4(p1433), P1433(dum~off,foxo4)
PDK1(PH!1), PIP-canon(three!1) → PDK1(PH), PIP-canon(three)
mTOR(sin!1,fkb12,heat), Rictor(sin!2,m2,m1), SIN1(ric!2,m!1) →
  mTOR(sin!3,fkb12!2,heat!1), Rictor(sin!4,m2!2,m1!1), SIN1(ric!4,m!3)
Akt-canon(T308~p,subs,S473~p), GSK3a(S21~u,a) →
  Akt-canon(T308~p,subs!1,S473~p), GSK3a(S21~u,a!1)
Akt-canon(foxo3a!1), FoxO3A(T32~u,akt!1) →
  Akt-canon(foxo3a!1), FoxO3A(T32~p,akt!1)
IR-canon(alpha), Insulin-canon(ir) →
  IR-canon(alpha!1), Insulin-canon(ir!1)
FKB12(m,rap!1), rapamycin(fkb12!1), mTOR(fkb12,l,heat,sin) →
  FKB12(m!2,rap!1), rapamycin(fkb12!1), mTOR(fkb12!2,l,heat,sin)
Akt-canon(foxo3a!1), FoxO3A(S253~u,akt!1) →
  Akt-canon(foxo3a!1), FoxO3A(S253~p,akt!1)
IR-canon(alpha!1), Insulin-canon(ir!1) →
  IR-canon(alpha), Insulin-canon(ir)
mTOR(l!1), mLST8(m!1) → mTOR(l), mLST8(m)
mTOR(sin!1), SIN1(m!1) → mTOR(sin), SIN1(m)
IR-canon(NPXY~p!1), IRS2(PTB!1,Y~u) → IR-canon(NPXY~p!1), IRS2(PTB!1,Y~p)
IGF1(igf1r), IGF1R(alpha) → IGF1(igf1r!1), IGF1R(alpha!1)
IGF1R(NPXY~p!1), IRS2(PTB!1,Y~u) → IGF1R(NPXY~p!1), IRS2(PTB!1,Y~p)
PTP1B(receptor!1), IGF1R(ptp!1) → PTP1B(receptor), IGF1R(ptp)
IRS2(Y~p,pi3k), PI3K-canon(p110~u,SH2-p85) →
  IRS2(Y~p,pi3k!1), PI3K-canon(p110~u,SH2-p85!1)
FoxO3A(S253~p,T32~p,p1433), P1433(dum~off,foxo3a) →
  FoxO3A(S253~p,T32~p,p1433!1), P1433(dum~on,foxo3a!1)
IGF1(igf1r!1), IGF1R(alpha!1) → IGF1(igf1r), IGF1R(alpha)
IR-canon(NPXY~p), IRS2(PTB,Y~u) → IR-canon(NPXY~p!1), IRS2(PTB!1,Y~u)
FoxO3A(p1433!1), P1433(dum~on,foxo3a!1) →
  FoxO3A(p1433), P1433(dum~off,foxo3a)
PKCz-canon(glut!1), Glut4-canon(pkc~off!1) →
  PKCz-canon(glut!1), Glut4-canon(pkc~on!1)
FoxO4(S193~u,T28~u,akt), Akt-canon(T308~p,foxo4,S473~p) →
  FoxO4(S193~u,T28~u,akt!1), Akt-canon(T308~p,foxo4!1,S473~p)
Akt-canon(foxo1!1), FoxO1(S256~p,T24~u,akt!1) →
  Akt-canon(foxo1!1), FoxO1(S256~p,T24~p,akt!1)
mTOR(heat!1,fkb12!2), Rictor(m2!2,m1!1) → mTOR(heat,fkb12), Rictor(m2,m1)
Glut4-canon(loc~gsv,akt~on,pkc~on) → Glut4-canon(loc~pm,akt~on,pkc~off)
IGF1R(NPXY~p), IRS2(PTB,Y~u) → IGF1R(NPXY~p!1), IRS2(PTB!1,Y~u)
Akt-canon(foxo1!1), FoxO1(S256~p,S319~u,akt!1) →
  Akt-canon(foxo1!1), FoxO1(S256~p,S319~p,akt!1)
Glut4-canon(rab!1,akt~off), Rab10(g~gtp!1) →
  Glut4-canon(rab!1,akt~on), Rab10(g~gtp!1)
FoxO3A(S253~u,T32~u,akt), Akt-canon(T308~p,foxo3a,S473~p) →
  FoxO3A(S253~u,T32~u,akt!1), Akt-canon(T308~p,foxo3a!1,S473~p)
PI3K-canon(SH2-p85!1), IRS2(pi3k!1) → PI3K-canon(SH2-p85), IRS2(pi3k)
GSK3a(S21~u,glysyn,a), GS(ST~p,STXXXST~u) →
  GSK3a(S21~u,glysyn!1,a), GS(ST~p,STXXXST~u!1)
GS(STXXXST!1), PP1(glysyn!1) → GS(STXXXST), PP1(glysyn)
```

```
SIN1(m!4,ric!3,akt!5), Akt-canon(S473~u!5), Rictor(sin!3,m2!2,m1!1),
  mLST8(m!6), mTOR(sin!4,heat!1,l!6,fkb12!2) ->
  SIN1(m!4,ric!3,akt!5), Akt-canon(S473~p!5),
  Rictor(sin!3,m2!2,m1!1), mLST8(m!6), mTOR(sin!4,heat!1,l!6,fkb12!2)
IR-canon(NPXY!1), IRS1-canon(PTB!1) -> IR-canon(NPXY), IRS1-canon(PTB)
Akt-canon(foxo4!1), FoxO4(akt!1) -> Akt-canon(foxo4), FoxO4(akt)
GSK3a(S21~p,glysyn!1,a!2), Akt-canon(subs!2), GS(STXXXST~p!1) ->
  GSK3a(S21~p,glysyn,a!1), Akt-canon(subs!1), GS(STXXXST~p)
SIN1(akt), Akt-canon(S473~u,T308~p) -> SIN1(akt!1), Akt-canon(S473~u!1,T308~p)
IGF1R(NPXY!1), IRS1-canon(PTB!1) -> IGF1R(NPXY), IRS1-canon(PTB)
CK2(glysyn!1), GS(ST~u!1) -> CK2(glysyn!1), GS(ST~p!1)
Akt-canon(T308~p), PP2A(B55-a) -> Akt-canon(T308~p!1), PP2A(B55-a!1)
IR-canon(NPXY~p,ptp!1,Y~p), PTP1B(receptor!1) ->
  IR-canon(NPXY~u,ptp!1,Y~u), PTP1B(receptor!1)
Akt-canon(foxo3a!1), FoxO3A(akt!1) -> Akt-canon(foxo3a), FoxO3A(akt)
Akt-canon(foxo1!1), FoxO1(akt!1) -> Akt-canon(foxo1), FoxO1(akt)
Akt-canon(PH), PIP-canon(three~p) -> Akt-canon(PH!1), PIP-canon(three~p!1)
GS(STXXXST~p!1), PP1(glysyn!1,ins~p) -> GS(STXXXST~u!1), PP1(glysyn!1,ins~p)
PKCz-canon(pdk1!1,T410~u), PDK1(pkc!1) ->
  PKCz-canon(pdk1!1,T410~p), PDK1(pkc!1)
AS160(gap!1), Akt-canon(as160!1) -> AS160(gap), Akt-canon(as160)
PHLPP(PDZ), Akt-canon(S473~p) -> PHLPP(PDZ!1), Akt-canon(S473~p!1)
GSK3b(glysyn!1), GS(STXXXST~u!1) -> GSK3b(glysyn!1), GS(STXXXST~p!1)
PKCz-canon(pdk1,T410~u), PDK1(pkc,PH) ->
  PKCz-canon(pdk1!1,T410~u), PDK1(pkc!1,PH)
AS160(gap~u!1), Akt-canon(as160!1) -> AS160(gap~p!1), Akt-canon(as160!1)
IR-canon(alpha,Y~u) -> IR-canon(alpha,Y~p)
GSK3a(glysyn!1), GS(STXXXST~u!1) -> GSK3a(glysyn!1), GS(STXXXST~p!1)
IR-canon(alpha,NPXY~u) -> IR-canon(alpha,NPXY~p)
PKCz-canon(pdk1!1), PDK1(pkc!1) -> PKCz-canon(pdk1), PDK1(pkc)
AS160(gap~u), Rab10(g~gtp) -> AS160(gap~u!1), Rab10(g~gtp!1)
IR-canon(NPXY~p,ptp,Y~p), PTP1B(receptor) ->
  IR-canon(NPXY~p,ptp!1,Y~p), PTP1B(receptor!1)
PIP-canon(three~p!1), PTEN(subs!1) -> PIP-canon(three~u!1), PTEN(subs!1)
AS160(gap!1), Rab10(g!1) -> AS160(gap), Rab10(g)
IR-canon(ptp!1), PTP1B(receptor!1) -> IR-canon(ptp), PTP1B(receptor)
PI3K-canon(p110~p,subs!1,SH2-p85), PIP-canon(three~u!1) ->
  PI3K-canon(p110~p,subs!1,SH2-p85), PIP-canon(three~p!1)
IGF1R(Y1136~u,alpha) -> IGF1R(Y1136~p,alpha)
IRS2(pi3k!1), PI3K-canon(p110~u,SH2-p85!1) ->
  IRS2(pi3k!1), PI3K-canon(p110~p,SH2-p85!1)
CK2(glysyn), GS(ST~u) -> CK2(glysyn!1), GS(ST~u!1)
IRS1-canon(pi3k!1), PI3K-canon(p110~u,SH2-p85!1) ->
  IRS1-canon(pi3k!1), PI3K-canon(p110~p,SH2-p85!1)
CK2(glysyn!1), GS(ST!1) -> CK2(glysyn), GS(ST)
IGF1R(Y1135~u,alpha) -> IGF1R(Y1135~p,alpha)
Akt-canon(PH!1,T308~u), PDK1(PH!2,akt),
  PIP-canon(three~p!2), PIP-canon(three~p!1) ->
  Akt-canon(PH!1,T308~u!2), PDK1(PH!3,akt!2),
  PIP-canon(three~p!3), PIP-canon(three~p!1)
PI3K-canon(p110~p,subs,SH2-p85), PIP-canon(three~u) ->
  PI3K-canon(p110~p,subs!1,SH2-p85), PIP-canon(three~u!1)
GEF(gef), Rab10(g~gdp) -> GEF(gef!1), Rab10(g~gdp!1)
mTOR(l), mLST8(m) -> mTOR(l!1), mLST8(m!1)
Rab10(g~gtp), Glut4-canon(loc~gsv,rab,akt~off) ->
  Rab10(g~gtp!1), Glut4-canon(loc~gsv,rab!1,akt~off)
FoxO1(S256~u,S319~u,T24~u,akt), Akt-canon(T308~p,foxo1,S473~p) ->
  FoxO1(S256~u,S319~u,T24~u,akt!1), Akt-canon(T308~p,foxo1!1,S473~p)
IR-canon(NPXY!1), IRS2(PTB!1) -> IR-canon(NPXY), IRS2(PTB)
Akt-canon(foxo1!1), FoxO1(S256~u,akt!1) ->
  Akt-canon(foxo1!1), FoxO1(S256~p,akt!1)
Glut4-canon(loc~gsv,pkc~off), PKCz-canon(glut,T410~p) ->
  Glut4-canon(loc~gsv,pkc~off!1), PKCz-canon(glut!1,T410~p)
FKB12(m!2), mTOR(fkb12!2) -> FKB12(m), mTOR(fkb12)
AS160(gap~u!1), Rab10(g~gdp!1), Akt-canon(as160,S473~p,T308~p) ->
  AS160(gap~u!1), Rab10(g~gdp), Akt-canon(as160!1,S473~p,T308~u)
FoxO1(S256~p,S319~p,T24~p,p1433), P1433(dum~off,foxo1) ->
  FoxO1(S256~u,S319~u,T24~u,p1433!1), P1433(dum~on,foxo1!1)
Glut4-canon(rab!1), Rab10(g~gtp!1) -> Glut4-canon(rab), Rab10(g~gtp)
SIN1(akt!1), Akt-canon(S473!1) -> SIN1(akt), Akt-canon(S473)
FoxO1(p1433!1), P1433(dum~on,foxo1!1) -> FoxO1(p1433), P1433(dum~off,foxo1)
```

```
PKCz-canon(glut!1), Glut4-canon(pkc!1) -> PKCz-canon(glut), Glut4-canon(pkc)
SIN1(ric), Rictor(sin) -> SIN1(ric!4), Rictor(sin!4)
Akt-canon(T308!1), PP2A(B55-a!1) -> Akt-canon(T308), PP2A(B55-a)
rapamycin(fkb12), FKB12(rap) -> rapamycin(fkb12!1), FKB12(rap!1)
IGF1R(NPXY!1), IRS2(PTB!1) -> IGF1R(NPXY), IRS2(PTB)
SIN1(ric!1), Rictor(sin!1) -> SIN1(ric), Rictor(sin)
Akt-canon(PH!1), PIP-canon(three!1) -> Akt-canon(PH), PIP-canon(three)
FKB12(rap!1), rapamycin(fkb12!1) -> FKB12(rap), rapamycin(fkb12)
IR-canon(NPXY~p!1), IRS1-canon(PTB!1,Y~u) ->
  IR-canon(NPXY~p!1), IRS1-canon(PTB!1,Y~p)
GEF(gef!1), Rab10(g~gdp!1) -> GEF(gef!1), Rab10(g~gtp!1)
PHLPP(PDZ!1), Akt-canon(S473!1) -> PHLPP(PDZ), Akt-canon(S473)
Akt-canon(T308~p!1), PP2A(B55-a!1) -> Akt-canon(T308~u!1), PP2A(B55-a!1)
IGF1R(NPXY~p!1), IRS1-canon(PTB!1,Y~u) ->
  IGF1R(NPXY~p!1), IRS1-canon(PTB!1,Y~p)
AS160(gap~u!1), Rab10(g~gtp!1) -> AS160(gap~u!1), Rab10(g~gdp!1)
Akt-canon(T308!1), PDK1(akt!1) -> Akt-canon(T308), PDK1(akt)
IRS1-canon(Y~p,pi3k), PI3K-canon(p110~u,SH2-p85) ->
  IRS1-canon(Y~p,pi3k!1), PI3K-canon(p110~u,SH2-p85!1)
PIP-canon(three~p), PTEN(subs) -> PIP-canon(three~p!1), PTEN(subs!1)
GSK3b(glysyn!1,a!2,S9~u), Akt-canon(subs!2), GS(STXXXST!1) ->
  GSK3b(glysyn!1,a!2,S9~p), Akt-canon(subs!2), GS(STXXXST!1)
Akt-canon(subs!1), GSK3b(a!1) -> Akt-canon(subs), GSK3b(a) @ 1.0
GSK3b(S9~u,glysyn,a), GS(ST~p,STXXXST~u) ->
  GSK3b(S9~u,glysyn!1,a), GS(ST~p,STXXXST~u!1)
IR-canon(NPXY~p), IRS1-canon(PTB,Y~u) ->
  IR-canon(NPXY~p!1), IRS1-canon(PTB!1,Y~u)
PIP-canon(three!1), PTEN(subs!1) -> PIP-canon(three), PTEN(subs)
GSK3b(glysyn!1,a!2,S9~p), Akt-canon(subs!2), GS(STXXXST~p!1) ->
  GSK3b(glysyn,a!1,S9~p), Akt-canon(subs!1), GS(STXXXST~p)
IGF1R(NPXY~p), IRS1-canon(PTB,Y~u) -> IGF1R(NPXY~p!1), IRS1-canon(PTB!1,Y~u)
Akt-canon(PH!1,T308~u!2), PDK1(PH!3,akt!2),
  PIP-canon(three~p!1), PIP-canon(three~p!3) ->
  Akt-canon(PH!1,T308~p!2), PDK1(PH!3,akt!2),
  PIP-canon(three~p!1), PIP-canon(three~p!3)
Akt-canon(T308~p,S473~p), PP1(ins~u) -> Akt-canon(T308~p,S473~p), PP1(ins~p)
Akt-canon(subs!1), GSK3a(a!1) -> Akt-canon(subs), GSK3a(a)
IRS1-canon(pi3k!1), PI3K-canon(SH2-p85!1) ->
  IRS1-canon(pi3k), PI3K-canon(SH2-p85)
PHLPP(PDZ!1), Akt-canon(S473~p!1) -> PHLPP(PDZ!1), Akt-canon(S473~u!1)
GS(STXXXST~p), PP1(glysyn,ins~p) -> GS(STXXXST~p!1), PP1(glysyn!1,ins~p)
Akt-canon(subs!2), GSK3a(glysyn!1,a!2,S21~u), GS(STXXXST!1) ->
  Akt-canon(subs!2), GSK3a(glysyn!1,a!2,S21~p), GS(STXXXST!1)
```
