# Supplementary material for: A High-Level Language for Rule-Based Modelling
Source: PLoS One. 2015 Jun 4;10(6):e0114296. doi: 10.1371/journal.pone.0114296 (PMC4456403; doi:10.1371/journal.pone.0114296)
Supplement: S4 Listing — (PDF) [file pone.0114296.s004.pdf]

Listing S4: A modular LBS-κ insulin signalling model.

```
// ----------------------------------------------------
// New agent definitions.
// ----------------------------------------------------
agent AS160 = new{gap};
agent Rab10 = new{g}; // we use u/p instead of gdp/gtp.
agent FoxO1 = new{S256, akt, T24, S319, p1433};
agent FoxO3A = new{S253, akt, p1433, T32};
agent FoxO4 = new{T28, akt, S193, p1433};
agent GSK3a = new{S21, a, glysyn};
agent GSK3b = new{a, glysyn, S9};
agent PDK1 = new{akt, PH, pkc};
agent PHLPP = new{PDZ};
agent PIP = new{three};
agent PP2A = new{B55a};
agent SIN1 = new{m, akt, ric};
agent mLST8 = new{m};
agent mTOR = new{l, heat, sin, fkb12};
agent FKB12 = new{m, rap};
agent rapamycin = new{fkb12};
agent P1433 = new{foxo3a, foxo4, foxo1, dum};
agent Glut4 = new{rab, loc:kappa(gsv pm), pkc, akt};
agent GEF = new{gef};
agent CK2 = new{glysyn};
agent GS = new{ST, STXXXST};
agent PP1 = new{ins, glysyn};
agent Rictor = new{m2, sin, m1};
agent PKCz = new{pdk1, glut, T410};
agent IGF1 = new{igf1r};
agent IGF1R = new{Y1131, Y1136, Y1135, alpha, NPXY, ptp};
agent PTP1B = new{receptor};
agent IR = new{alpha, Y, NPXY, ptp};
agent IRS1 = new {Y, PTB, PH, pi3k};
agent IRS2 = new {Y, PTB, PH, pi3k};
agent PTEN = new{subs};
agent PI3K = new{p110, subs, SH2p85};
agent Insulin = new{ir};
agent Akt = new{foxo3a, foxo4, foxo1, subs, as160, PH, T308, S473};

// ----------------------------------------------------
// Some single-rule utility modules.
// ----------------------------------------------------

// a module for binding two agents.
module bind( agent a:{m}, b:{m} ) {
  a{m} + b{m} → a{m!1}-b{m!1}
};

// a module for unbinding two agents.
module unbind( agent a:{m}, b:{m} ) {
  a{m!1}-b{m!1} → a{m} + b{m}
};

// a module for phosphorylating an agent with given binding.
module pho( agent a:{m} ) {
  a{m~u!e} → a{m~p!e}
};

// a module for phosphorylating an agent with no binding.
module pho2( agent s:{m} ) {
    pho(s{m}:{m})
};

// a module for dephosphorylating an agent.
module depho( agent a:{m} ) {
  a{m~p!e} → a{m~u!e}
};

// ----------------------------------------------------
// Modules for multi-rule phosphorylation.
// ----------------------------------------------------
```

```
// a general module for binding, phos and unbinding, where
// agent components are provided for individual reactions.
// binding and phosphorylation is modelled here on separate
// sites, but can take place on the same site by instantiating
// both s2 actuals to the same.
module bpu0( agent k1:{m}, k2:{m}, k3:{m}, s1:{m1, m2}, s2:{m1, m2}, s3:{m} ) {
  k1{m} + s1{m1~u!e, m2} → k1{m!1}-s1{m1~u!e, m2!1} |
  k2{m!1}-s2{m1~u!e, m2!1} → k2{m!1}-s2{m1~p!e, m2!1} |
  k3{m!1}-s3{m!1} → k3{m} + s3{m}
};

// plain bind-phosphorylate-unbind, all on same site.
module bpu( agent k:{m}, s:{m} ) {
  bpu0( k:{m}, k:{m}, k:{m}, s:{m, m}, s:{m, m}, s:{m} )
};

// a module to bind-phophorylate-unbind; binding on second site of substrate.
// kinase is phosphorylated on binding site in first two rules.
module bpu2( agent k:{m}, s:{m1,m2} ) {
  bpu0(
    k{m~p}:{m},     k{m~p}:{m},     k:{m},
    s{m1}:{m1,m2}, s{m1}:{m1,m2}, s:{m2}
  )
};

// a module to bind-phophorylate-unbind; binding on second site of substrate.
// kinase specifies an additional site in first rule only.
module bpu3( agent k:{m1, m2}, s:{m1,m2} ) {
  bpu0(
    k:{m2},         k{m1?}:{m2},    k{m1?}:{m2},
    s{m1}:{m1,m2}, s{m1}:{m1,m2}, s:{m2}
  )
};

// a module to bind-phosphorylate-phosphorylate-unbind;
// substrate sites m1 and m2 are modified, binding on m3.
// kinase is phosphorylated on first two sites in binding rule.
module bppu( agent k:{m1,m2,m3}, s:{m1, m2, m3} ) {
  bind( k{m1~p, m2~p, m3}:{m3}, s{m1~u, m2~u, m3}:{m3}) |
  pho(k{m3!1}-s{m1~u, m3!1}:s{m1}) |
  pho(k{m3!1}-s{m2~u, m3!1}:s{m2}) |
  unbind( k:{m3}, s:{m3} )
};

// ---------------------------------------------------
// Modules for multi-rule dephosphorylation
// ---------------------------------------------------

// a general module for binding, dephos and unbinding, where
// agent components are provided for individual reactions.
// binding and phosphorylation is modelled here on separate
// sites, but can take place on the same site by instantiating
// both s2 actuals to the same.
module bdu0( agent p1:{m}, p2:{m}, p3:{m}, s1:{m1, m2}, s2:{m1, m2}, s3:{m} ) {
  p1{m} + s1{m1~p!e, m2} → p1{m!1}-s1{m1~p!e, m2!1} |
  p2{m!1}-s2{m1~p!e, m2!1} → p2{m!1}-s2{m1~u!e, m2!1} |
  p3{m!1}-s3{m!1} → p3{m} + s3{m}
};

// a plain module to bind-dephos-unbind, all on same site.
module bdu( agent ph:{m}, s:{m} ) {
  bdu0( ph:{m}, ph:{m}, ph:{m}, s:{m, m}, s:{m, m}, s:{m} )
};

// a module to bind-dephos-unbind, all on same site,
// but with phosphatase unphos in first two rules.
module bdu2( agent ph:{m}, s:{m} ) {
  bdu0( ph{m~u}:{m}, ph{m~u}:{m~u}, ph:{m}, s:{m, m}, s:{m, m}, s:{m} )
};
```

```
// a module to bind-dephos-unbind, with an additional site in phosphatase.
module bdu3( agent  ph:{m1, m2}, s:{m} ) {
  bdu0( ph{m2~p}:{m1}, ph{m2~p}:{m1}, ph:{m1}, s:{m, m}, s:{m, m}, s:{m} )
};

// a module to bind, dephos on four sites, and unbind.
// binding takes place on distint site (last substrate site).
// fewer sites can be dephosphorylated by repeating actual parameters.
module bddddu( agent  ph:{m}, s:{m1,m2,m3,m4,m0} ) {
  ph{m} + s{m1~p, m2~p, m3~p, m4~p, m0} → ph{m!1}-s{m1~p, m2~p, m3~p, m4~p, m0!1} |
  ph{m!1}-s{m1~p, m2~p, m3~p, m4~p, m0!1} → ph{m!1}-s{m1~u, m2~u, m3~u, m4~u, m0!1} |
  ph{m!1}-s{m0!1} → ph{m} + s{m0}
};

// a module to bind, and with dephos/unbind in same rule.
module bd( agent ph:{m1, m2, m3}, s:{m1, m2} ) {
  ph{m1~p, m2~p, m3} + s{m1~u, m2} →  ph{m1~p, m2~p, m3!1}-s{m1~p, m2!1} |
  ph{m3!1}-s{m1~p, m2!1} → ph{m3} + s{m1~u, m2}
};

// ----------------------------------------------------
// Modules for binding and unbinding.
// ----------------------------------------------------

// a bind-unbind module; site on b is phosphorylated for binding.
module bu( agent a:{x}, b:{y} ) {
  a{x} + b{y~p} → a{x!1}-b{y~p!1} |
  a{x!1}-b{y!1} → a{x} + b{y}
};

// a bind-unbind module with additional state on both agents.
module bu2( agent a:{x, y, z}, b:{x, y} ) {
  a{x~u, y, z} + b{x~p, y~u} → a{x~u, y, z!1}-b{x~p, y~u!1} |
  a{z!1}-b{y~u!1} → a{z!1}-b{y~p!1}
};

// two non-deterministic agents.
agent GSK3 = (GSK3b:{glysyn, a, S9} or GSK3a:{glysyn, a, S21}) :: GSK3{glysyn, a, S};
agent IRS = (IRS1:{PTB, Y, pi3k} or IRS2:{PTB, Y, pi3k}) :: IRS{PTB, Y, pi3k};

// ----------------------------------------------------
// Modules defining key components of the system.
// ----------------------------------------------------
module receptorActivation() {
  bpu2(IGF1R:{NPXY}, IRS:{Y, PTB}) |
  bpu2(IR:{NPXY}, IRS:{Y, PTB}) |
  IGF1R{alpha} + IGF1{igf1r} ⟨→ IGF1R{alpha!1}-IGF1{igf1r!1} |
  IR{alpha} + Insulin{ir} ⟨→ IR{alpha!1}-Insulin{ir!1} |

  pho2(IGF1R{alpha}:{Y1131}) |
  pho2(IGF1R{alpha}:{NPXY}) |
  pho2(IGF1R{alpha}:{Y1136}) |
  pho2(IGF1R{alpha}:{Y1135}) |
  pho2(IR{alpha}:{Y}) |
  pho2(IR{alpha}:{NPXY}) |

  bddddu( PTP1B:{receptor}, IGF1R:{Y1131, Y1136, Y1135, NPXY, ptp} ) |
  bddddu( PTP1B:{receptor}, IR:{NPXY, Y, Y, Y, ptp} )
};

module pipAktSignalling() {
  module pipSignalling() {
    bpu3( IRS{Y~p}:{Y, pi3k}, PI3K:{p110, SH2p85} ) |
    bpu0(
      PI3K{p110~p, subs, SH2p85}:{subs},
      PI3K{p110~p, subs, SH2p85}:{subs},
      PI3K{subs}:{subs},
      PIP:{three, three},
      PIP:{three, three},
      PIP:{three}
    ) |
```

```
    bdu( PTEN:{subs}, PIP:{three} ) |

    bu(PDK1:{PH}, PIP:{three}) |
    bu(Akt:{PH}, PIP:{three})
  };

  module aktSignalling() {
    Akt{PH!1, T308~u}-PIP{three~p!1} as a + PDK1{PH!2, akt}-PIP{three~p!2} as b → a⟨Akt{T308~e!4}⟩-b⟨PDK1{akt!4}⟩ as c;
    c → c⟨Akt{T308~p!e}⟩ |
    Akt{T308!1}-PDK1{akt!1} → Akt{T308} + PDK1{akt} |

    bind(  SIN1:{akt}, Akt{S473~u, T308~p}:{S473} ) |
    SIN1{m!4, ric!3, akt!5}-Akt{S473~u!5}-Rictor{sin!3, m2!2, m1!1}-mLST8{m!6}-mTOR{sin!4, heat!1, l!6, fkb12!2} as c →{33} c⟨Akt{S473~p!e}⟩ |
    unbind( SIN1:{akt},  Akt:{S473} ) |

    bdu(PP2A:{B55a}, Akt:{T308}) |
    bdu(PHLPP:{PDZ}, Akt:{S473})
  };

  pipSignalling() |
  aktSignalling()
};

module gluconeogenesis() {
  bppu(Akt:{T308, S473, foxo4}, FoxO4:{T28, S193, akt} ) |
  bppu(Akt:{T308, S473, foxo3a}, FoxO3A:{T32, S253, akt} ) |

  // phosphorylation of Fox01 by Akt is similar to the above, but
  // there is an additional site which is phosphorylated in an
  // additional rule.
  bind( Akt{T308~p, foxo1, S473~p}:{foxo1}, FoxO1{S256~u, S319~u, T24~u, akt}:{akt} ) |
  pho( Akt{foxo1!1}-FoxO1{S256~u, akt!1}: FoxO1{S256} ) |
  pho( Akt{foxo1!1}-FoxO1{S256~p, T24~u, akt!1}: FoxO1{T24} ) |
  pho( Akt{foxo1!1}-FoxO1{S256~p, S319~u, akt!1}: FoxO1{S319} ) |
  unbind( Akt:{foxo1}, FoxO1:{akt} ) |

  bd(FoxO4:{S193, T28, p1433}, P1433:{dum, foxo4}) |
  bd(FoxO3A:{S253, T32, p1433}, P1433:{dum, foxo3a}) |
  // the following are similar, but also dephosphorylates kinase!
  FoxO1{S256~p, S319~p, T24~p, p1433} + P1433{dum~u, foxo1} → FoxO1{S256~u, S319~u, T24~u, p1433!1}-P1433{dum~p, foxo1!1} |
  FoxO1{p1433!1}-P1433{dum~p, foxo1!1} → FoxO1{p1433} + P1433{dum~u, foxo1}
};

module glycogenSynthesis() {
  bu2(GSK3:{S, a, glysyn},  GS:{ST, STXXXST}) |

  bind( Akt{T308~p, subs, S473~p}:{subs}, GSK3{S~u, a}:{a}) |
  pho( Akt{subs!2}-GSK3{glysyn!1, a!2, S~u}-GS{STXXXST!1}: GSK3{S} ) |
  unbind( Akt:{subs}, GSK3:{a} ) |

  Akt{subs!2}-GSK3{glysyn!1, a!2, S~p}-GS{STXXXST~p!1} → Akt{subs!1}-GSK3{glysyn, a!1, S~p} + GS{STXXXST~p} |

  bdu3(PP1:{glysyn, ins}, GS:{STXXXST})
};

module cellGrowth() {
  mTOR{sin} + SIN1{m} ⟨→ mTOR{sin!1}-SIN1{m!1} |
  mTOR{l} + mLST8{m} ⟨→ mTOR{l!1}-mLST8{m!1} |

  SIN1{ric} + Rictor{sin} ⟨→ SIN1{ric!4}-Rictor{sin!4} |
  FKB12{rap} + rapamycin{fkb12} ⟨→ FKB12{rap!1}-rapamycin{fkb12!1} |

  // two more bindings between mtor and rictor:
  mTOR{sin!1, fkb12, heat}-Rictor{sin!2, m2, m1}-SIN1{ric!2, m!1} as c → c⟨mTOR{fkb12!4, heat!3}⟩⟨Rictor{m2!4, m1!3}⟩ |
  mTOR{heat!1, fkb12!2}-Rictor{m2!2, m1!1} → mTOR{heat, fkb12} + Rictor{m2, m1} |

```

```
  bind(FKB12{m, rap!1}-rapamycin{fkb12!1}: FKB12{m}, mTOR{fkb12, l, heat, sin}:{fkb12} ) |
  unbind( FKB12:{m}, mTOR:{fkb12} )
};

module glucoseUptake() {
  bpu3( PDK1{PH}:{PH, pkc}, PKCz:{T410, pdk1} ) |

  Akt{as160, S473~p, T308~p} + AS160{gap~u!1}-Rab10{g~u!1} → Akt{as160!1, S473~p, T308~u}-AS160{
      gap~u!1} + Rab10{g~u} |
  pho( Akt{as160!1}-AS160{gap~u!1}: AS160{gap}) |
  unbind( Akt:{as160}, AS160:{gap} ) |

  Akt{T308~p, S473~p} + PP1{ins~u} → Akt{T308~p, S473~p} + PP1{ins~p} |

  bind( PKCz{glut, T410~p}:{glut}, Glut4{loc~gsv, pkc~u}:{pkc} ) |
  pho(  PKCz{glut!1}-Glut4{pkc~u!1}: Glut4{pkc} ) |
  unbind( PKCz:{glut}, Glut4:{pkc} ) |

  bind( Rab10{g~p}:{g}, Glut4{loc~gsv, rab, akt~u}:{rab} ) |
  pho(  Rab10{g~p!1}-Glut4{rab!1, akt~u}: Glut4{akt} ) |
  unbind( Rab10{g~p}:{g}, Glut4:{rab} ) |

  bpu(CK2:{glysyn}, GS:{ST}) |

  bpu(GEF:{gef}, Rab10:{g}) |
  bdu2(AS160:{gap}, Rab10:{g}) |

  Glut4{loc~gsv, akt~p, pkc~p} → Glut4{loc~pm, akt~p, pkc~u}
};

// ---------------------------------------------------
// Main body of model with module instantiations.
// ---------------------------------------------------
receptorActivation() |
pipAktSignalling() |
gluconeogenesis() |
glycogenSynthesis() |
cellGrowth() |
glucoseUptake()
```
