# Supplementary material for: A High-Level Language for Rule-Based Modelling
Source: PLoS One. 2015 Jun 4;10(6):e0114296. doi: 10.1371/journal.pone.0114296 (PMC4456403; doi:10.1371/journal.pone.0114296)
Supplement: S5 Listing — Included for comparison with the LBS-κ MAPK cascade model. Lines 1-15 define the cycle module. Lines 17-25 define the agents, here called monomers. Lines 27-31 contain the module instantiations. Each of these three blocks have similar blocks in the corresponding LBS-κ model. Because PySB distinguishes between sites for binding and sites with internal states, some sites are separated into two in the PySB model; we use the “b”-prefix for sites which are used for binding, and the “m”-prefix for sites which have modification state. The syntax of PySB is otherwise inherited from Python, a detailed description of which is outside the scope of this comparison. We note that, although we have attempted to construct a model which is equivalent to the LBS-κ model, we have not been able to verify that the the models do indeed translate to equivalent flat models. (PDF) [file pone.0114296.s005.pdf]

Listing S5: An example PySB model of a MAPK cascade. Included for comparison with the LBS-$\kappa$ MAPK cascade model. Lines 1-15 define the cycle module. Lines 17-25 define the agents, here called *monomers*. Lines 27-31 contain the module instantiations. Each of these three blocks have similar blocks in the corresponding LBS-$\kappa$ model. Because PySB distinguishes between sites for binding and sites with internal states, some sites are separated into two in the PySB model; we use the "b"-prefix for sites which are used for binding, and the "m"-prefix for sites which have modification state. The syntax of PySB is otherwise inherited from Python, a detailed description of which is outside the scope of this comparison. We note that, although we have attempted to construct a model which is equivalent to the LBS-$\kappa$ model, we have not been able to verify that the the models do indeed translate to equivalent flat models.

```
1  Parameter('r', 1.0)
2  Parameter('I', 10)
3  def cycle(k, p, s, b1, b2, b3, m3, mid):
4      def bpu(k, s, b1, b2, m2, mid):
5          K = k({b1:None})
6          S = s({b2:None, m2:'u'})
7          S2 = s({b2:None, m2:'p'})
8          KS = k({b1:1}) % s({b2:1, m2: 'u'})
9          KS2 = k({b1:1}) % s({b2:1, m2: 'p'})
10         Rule(mid+'_bind', K + S >> KS, r)
11         Rule(mid+'mod', KS >> KS2, r)
12         Rule(mid+'unbind', KS2 >> K + S2, r)
13
14     def bdu(p, s, b1, b2, m2, mid):
15         P = p({b1:None})
16         S = s({b2:None, m2:'p'})
17         S2 = s({b2:None, m2:'u'})
18         PS = p({b1:1}) % s({b2:1, m2: 'p'})
19         PS2 = p({b1:1}) % s({b2:1, m2: 'u'})
20         Rule(mid+'_bind', P + S >> PS, r)
21         Rule(mid+'mod', PS >> PS2, r)
22         Rule(mid+'unbind', PS2 >> P + S2, r)
23
24     bpu(k, s, b1, b3, m3, mid+'_p')
25     bdu(p, s, b2, b3, m3, mid+'_u')
26
27 Monomer('Ras', ['b', 'm'], {'m':['gdp', 'gtp']})
28 Monomer('Raf', ['b', 'm'], {'m':['u', 'p']})
29 Monomer('MEK',
30     ['b_S218', 'm_S218', 'b_S222', 'm_S222', 'b'], {'m_S218':['u', 'p'], 'm_S222':['u', 'p']})
31 Monomer('ERK',
32     ['b_T185', 'm_T185', 'b_Y187', 'm_Y187'], {'m_T185':['u', 'p'], 'm_Y187':['u', 'p']})
33 Monomer('PP2A1', ['b'])
34 Monomer('PP2A2', ['b'])
35 Monomer('MKP3', ['b'])
36
37 cycle(Ras({'m':'gtp'}), PP2A1, Raf, 'b', 'b', 'b', 'm', 'module1')
38 cycle(Raf({'m':'p'}), PP2A2, MEK, 'b', 'b', 'b_S222', 'm_S222', 'module2')
39 cycle(Raf({'m':'p'}), PP2A2, MEK, 'b', 'b', 'b_S218', 'm_S218', 'module3')
40 cycle(MEK({'m_S218':'p', 'm_S222':'p'}), MKP3, ERK, 'b', 'b', 'b_T185', 'm_T185', 'module4')
41 cycle(MEK({'m_S218':'p', 'm_S222':'p'}), MKP3, ERK, 'b', 'b', 'b_Y187', 'm_Y187', 'module5')
```
